# The Relationship between Circadian Rhythm and Cancer Disease

**DOI:** 10.3390/ijms25115846

**Published:** 2024-05-28

**Authors:** Camelia Munteanu, Sabina Turti, Larisa Achim, Raluca Muresan, Marius Souca, Eftimia Prifti, Sorin Marian Mârza, Ionel Papuc

**Affiliations:** 1 Department of Plant Culture, Faculty of Agriculture, University of Agricultural Sciences and Veterinary Medicine Cluj-Napoca, Calea Mănăştur 3-5, 400372 Cluj-Napoca, Romania; camelia.munteanu@usamvcluj.ro (C.M.); alexandra.sabina.turti@student.usamvcluj.ro (S.T.); larisa-daniela.achim@student.usamvcluj.ro (L.A.); raluca-maria.muresan@student.usamvcluj.ro (R.M.); marius-viorel.souca@student.usamvcluj.ro (M.S.); eftimia.prifti@student.usamvcluj.ro (E.P.); 2 Faculty of Veterinary Medicine, University of Agricultural Sciences and Veterinary Medicine Cluj-Napoca, Calea Mănăştur 3-5, 400372 Cluj-Napoca, Romania; ionel.papuc@usamvcluj.ro

**Keywords:** circadian rhythm, cancer, melatonin, suprachiasmatic nucleus

## Abstract

The circadian clock regulates biological cycles across species and is crucial for physiological activities and biochemical reactions, including cancer onset and development. The interplay between the circadian rhythm and cancer involves regulating cell division, DNA repair, immune function, hormonal balance, and the potential for chronotherapy. This highlights the importance of maintaining a healthy circadian rhythm for cancer prevention and treatment. This article investigates the complex relationship between the circadian rhythm and cancer, exploring how disruptions to the internal clock may contribute to tumorigenesis and influence cancer progression. Numerous databases are utilized to conduct searches for articles, such as NCBI, MEDLINE, and Scopus. The keywords used throughout the academic archives are “circadian rhythm”, ”cancer”, and ”circadian clock”. Maintaining a healthy circadian cycle involves prioritizing healthy sleep habits and minimizing disruptions, such as consistent sleep schedules, reduced artificial light exposure, and meal timing adjustments. Dysregulation of the circadian clock gene and cell cycle can cause tumor growth, leading to the need to regulate the circadian cycle for better treatment outcomes. The circadian clock components significantly impact cellular responses to DNA damage, influencing cancer development. Understanding the circadian rhythm’s role in tumor diseases and their therapeutic targets is essential for treating and preventing cancer. Disruptions to the circadian rhythm can promote abnormal cell development and tumor metastasis, potentially due to immune system imbalances and hormonal fluctuations.

## 1. Introduction

Homeostasis in the human body is maintained by the complex and important interconnection between different systems in our body and the circadian cycle. This whole mechanism plays a major role in synchronizing our bodies with the natural rhythms of day and night [1]. However, recent research revealed that there is a strong connection between disruptions to the circadian rhythm and the development of cancer. Cancer is known to be one of the most fearful affections in the present [2]. Cancer diseases are a large and heterogeneous group of malignant tumors, defined by the uncontrollable proliferation of cells and their capability to spread in the whole human body [3]. In cancerous cells, significant changes occur in the activity of signaling pathways, affecting a wide range of cellular activities ranging from growth and proliferation to apoptosis, invasiveness, and metastasis [4]. Research indicated a familial coaggregation of breast and prostate cancer across various ethnic groups, suggesting a potential genetic predisposition alongside hormonal influences. Shared genetic abnormalities might play a role in the malignant transformation of mammary and prostatic epithelial cells. Furthermore, lifestyle and environmental factors, including dietary habits, could also contribute to the occurrence of these common cancers [5]. Colon and rectal cancer, like many epithelial neoplasms, exhibit a rise in occurrence with advancing age. While variations in the incidence exist within countries, they are generally overshadowed by more significant differences observed between nations. The dietary habits prevalent in Western cultures, characterized by refined protein-rich and high-fat foods, have been singled out as a potential factor contributing to the elevated rates of colon and rectal cancer in these regions [6]. During a normal circadian cycle, the immune system displays a biphasic shift, causing cytokines to be responsible for normal cellular functioning. Cytokines are small proteins that cells secrete, and they play a key role in how cells interact and communicate with each other [7]. When this rhythm deviates from its normal functioning path, cytokines begin to be produced excessively, resulting in immune system disturbances. These kinds of disturbances are known to be responsible for triggering tumors. Analyzing the unique cytokine profiles found in the tumor microenvironment can offer valuable insights into cancer detection, prognostication, and treatment decisions. By examining circulating cytokines alongside cancer-related parameters, we can enhance our ability to diagnose cancers, predict disease progression, and optimize therapeutic strategies [8]. Also, abnormal sleep–wake cycle fluctuations influence the production of melatonin, which is directly responsible for regulating the expression of certain genes to prevent tumor cell formation [9]. Understanding this specific relationship could lead to improved treatment approaches and offer new opportunities for treating cancer.

However, there remains a noticeable gap in the literature concerning the intricate interplay between the circadian rhythm and cancer. Despite substantial research in both fields, a significant inconsistency persists in our understanding of how circadian disruptions contribute to cancer development, progression, and treatment outcomes. This gap underscores the need for further investigation to elucidate the complex relationship between circadian biology and cancer pathology.

## 2. Methods

This article investigates the complex relationship between the circadian rhythm and cancer, exploring how disruptions to the internal clock may contribute to tumorigenesis and influence cancer progression. Circadian rhythm disruption in patients with cancer is examined to pinpoint any gaps in the specific literature. The primary research question is as follows: "What is known about circadian rhythm disruption in cancer patients in terms of its (1) elements, (2) frequency, (3) related factors, and (4) results?"

We utilized computer assistance to search many databases for articles: Web of Science, MEDLINE, NCBI, CINAHL, Cochrane Library, and Scopus. The following search terms were used: circadian rhythm, neoplasms/(cancer, tumor, neoplasm, malignant) and (advanced or metastasis or metastatic). All the authors searched relevant studies, which were included if they were scientific articles and written in English. Regarding the publishing year, there were no limitations. An additional manual search was carried out using the reference lists of relevant papers.

## 3. How Circadian Physiology Works 

Circadian clocks represent an evolutionarily conserved mechanism operating at the molecular level to temporally regulate physiological processes, thereby maintaining internal homeostasis. This regulatory system plays a pivotal role in orchestrating numerous vital biological functions. The circadian clock comprises two primary components: the central clock, housed within the brain suprachiasmatic nucleus (SCN), and peripheral clocks distributed throughout various tissues and organ systems. External indicators, such as light signals and feeding rhythms, entrain the central circadian clock, which subsequently synchronizes the peripheral clocks [10]. The molecular mechanism of the circadian clock can be described by the Transcription–Translation Oscillator (TTO) loop.

Comprising three fundamental elements—inputs, the oscillator, and outputs—the circadian system operates on both the systemic and cellular levels [11]. In mammals, the suprachiasmatic nucleus (SCN) situated within the hypothalamus serves as the "master clock," synchronizing with the environmental light–dark cycle by integrating signals from the autonomic nervous system’s efferent and neuroendocrine pathways. During the sleep–wake cycle, the immune system undergoes a biphasic shift in activity tied to the T-helper cell cytokine balance of T-helper1 (Th1) and T-helper2 (Th2) [12]. Th1 cytokines (e.g., IL-2, IFNγ, and IL-12) persist in the early hours of the night, while Th2 cytokines (e.g., IL-4 and IL-10) become more prominent in the late stages of sleep or just before awakening. This temporal shift entails an initial augmentation of Th1 activity during the early sleep phase, followed by a moderate increase in IFN-γ/IL-4-producing Th cells, culminating in the predominance of Th2 activity in the latter stages of sleep. Disruption of this pattern results in dysregulated cytokine production, leading to immune disturbances, chronic inflammation, and tissue damage [13].

The homeostatic physiology of the circadian rhythm, or the sleep–wake cycle, controls sleep. The alternations in an organism’s body, mind, and behavior that occur over 24 h are known as circadian rhythms. Circadian rhythms are primarily influenced by light and dark, but they are also influenced by temperature, food consumption, stress, physical activity, and social surroundings [14]. Important bodily functions are impacted by circadian rhythms, including the sleep–wake cycle, hormone secretion, heart rate, blood flow to the kidneys, immune system reaction to antigens, and many more. Therefore, an endogenous timing system is required to regulate these circadian variations or cycles [15].

**Figure 1:**
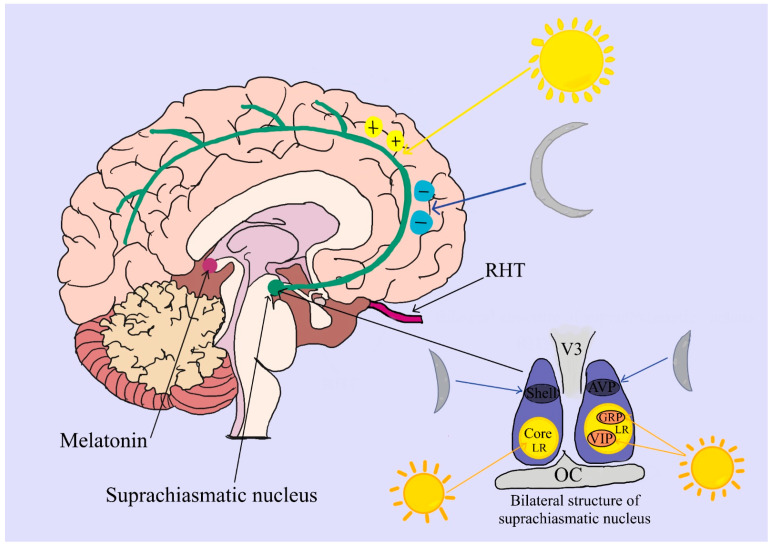
The connection between melatonin secretion and circadian rhythm. The suprachiasmatic nuclei (SCN), which are located immediately above the optic chiasm in the anterior-ventral area of the hypothalamus, are believed to include the pacemaker of this clock. This circadian clock reset comes about through the retinohypothalamic tract (RHT), which sends light information directly from the retina to a subset of SCN neurons. A pineal hormone known as melatonin is most abundant in the blood at night and least prevalent during the day. Its secretion is governed by a rhythm-generating process in the SCN, which is regulated by light. Melatonin is not only regulated by the circadian oscillator but also provides the oscillator with a feedback signal for darkness. Bilateral structure of the SCN and its “core” and “shell” subregions with vasoactive intestinal peptide (VIP) and gastrin-releasing peptide (GRP) in the light-responsive core and arginine vasopressin (AVP)-expressing cells in the shell; optic chiasm (OC); the 3rd cerebral ventricle (V3).

The circadian clock’s pacemaker is assumed to be situated in the suprachiasmatic nuclei (SCN). They are in the anterior-ventral region of the hypothalamus, directly above the optic chiasm [16]. The retinohypothalamic tract (RHT), which sends light information from the retina directly to a subset of SCN neurons, is responsible for this circadian clock reset [15]. Axons from the retinal ganglionic cells send impulses to the optic nerve, or cranial nerve II, during the light cycle, activating the suprachiasmatic nucleus (Figure 1).

The influence of the paraventricular nucleus is subsequently inhibited by a signal sent by the SCN via the inhibitory neurotransmitter gamma-amino-butyric acid (GABA). The sympathetic nervous system is then inhibited by axons that convey impulses from the intermediate lateral column to the superior cervical ganglion [17] Axons from the paraventricular nucleus are sent through the intermediolateral nucleus (IML) to the superior cervical ganglion, where they stimulate the sympathetic nervous system and cause drowsiness [18]. As night falls, the retinal ganglion cells receive a signal to inhibit the SCN, which activates the paraventricular nucleus. Melatonin is secreted into the bloodstream by the activation of the pineal gland [19]. 

Melatonin, a pineal hormone, is highest in the blood at night and lowest during the day. A rhythm-generating mechanism in the SCN controls its secretion and lightly regulates this system. In addition to being controlled by the circadian oscillator, melatonin also serves as a feedback signal for darkness, feeding back into the oscillator (Figure 1) [20]. Melatonin can synchronize the circadian cycle and have a soporific effect. It also plays a significant role in controlling the body temperature rhythm [21]. Many abnormalities of the circadian rhythm affect the melatonin rhythms. Treatment with melatonin is beneficial in treating conditions such as delayed sleep phase syndrome and jet lag [22].

The circadian activity of the SCN directly impacts the rhythmic secretion of numerous other hormones. The SCN directly regulates the secretion of vasopressin (AVP) into the cerebrospinal fluid. AVP acts as both a neurotransmitter within the SCN and an autocrine regulator, pacing neuronal activity. Acetylcholine (ACh) is among the neurotransmitters implicated in circadian rhythmicity and is released during wakefulness. Additionally, the SCN coordinates the release of glucocorticoid hormones, including adrenocorticotropic hormone (ACTH) and cortisol. Circadian rhythms also influence the secretion of insulin, ghrelin, leptin, and adiponectin [1].

The physiological responses of SCN cells to light primarily begin with glutamate activating the NMDA receptors in retinorecipient cells. This typically enhances the firing rate of these cells, triggering multiple intracellular cascades. These include elevations in the intracellular calcium levels, activation of various kinases, and phosphorylation of CREB, all contributing to the regulation of gene expression induced by light [23].

A strong correlation exists between the endogenous circadian component of the sleep propensity rhythm and the endogenous melatonin rhythm. Melatonin administration appears to have the following effects: (i) suppresses the circadian pacemaker’s drive for wakefulness; (ii) induces sleep when the homeostatic drive to sleep is insufficient; and (iii) causes phase shifts in the circadian clock, causing the circadian phase of increased sleep propensity to occur at a new, desired time. Thus, exogenous melatonin has the potential to function as a chronobiotic, a chronohypnotic, or a soporific [24].

Interestingly, the majority of completely blind individuals have irregular circadian rhythms [25]. They may not be able to engage in photic entrainment due to aberrant retinal processing and/or a malfunctioning RHT. Their circadian rhythms—which include the regularity of melatonin production—should thus have a pattern other than a 24-h cycle under this situation (Figure 1). It has been shown that the blind may experience free-running cycles for temperature, cortisol, melatonin, and, to a lesser extent, sleep. Studies have identified four distinct groups among blind individuals based on their circadian rhythms: (1) those who are regularly entrained to a 24-h cycle; (2) those with irregular entrainment to a 24-h cycle; (3) individuals exhibiting free-running rhythms lasting 24 h or less, and (4) those classified as unstable, displaying no discernible pattern in their circadian rhythms [26].

Many hormones have different levels throughout the day and at night. Certain hormones, including Growth Hormone (GH), are significantly impacted by sleep, whereas the circadian-timing system regulates other hormones more strongly [27]. The following factors affect GH secretion: body composition, fitness, sleep patterns, age, gender, and sex steroid hormones. Over a day, spontaneous GH secretion peaks at night and is closely linked to slow-wave sleep. At night, especially during slow-wave sleep, the GH response to an intravenous bolus injection of GHRH is at its highest. Given that GH secretion peaks at night when people are kept awake, circadian rhythms may have an independent effect on GH secretion from sleep [28]. Moreover, cortisol secretion pulses. A circadian rhythm controls the amplitude and frequency of these cortisol secretory pulses. Because of an increase in the frequency and amplitude of the cortisol secretory pulses, circulating cortisol concentrations are at their highest in the early morning hours right before waking. Throughout the day, the cortisol secretory pulse amplitude gradually drops until nightfall, when the cortisol concentrations are relatively low [29]. Contrary to nocturnal sleep, diurnal sleep was unable to inhibit the release of cortisol, demonstrating that sleep only inhibits cortisol release within a particular spectrum of entrainment instead of at any stage of the circadian rhythm [30]. 

Thus, the circadian rhythm is important in maintaining the body’s homeostasis. In general, maintaining good circadian rhythms is crucial for maintaining a general physiological and metabolic balance due to the complex interactions between the circadian timing system, sleep–wake cycles, and hormone control. Knowing these connections can help guide treatments that address hormone imbalances and circadian disturbance, eventually leading to improved health and well-being.

A class of genes known as lock genes encode proteins that are part of the molecular machinery of the circadian rhythms, which are internal biological clocks that control physiological activities that occur every day. These genes are essential for coordinating the cyclical expression of many downstream target genes related to hormone secretion, metabolism, and other oscillatory daily processes in cells [31,32,33]. Clock genes control the body’s circadian rhythm through complicated biochemical processes. Transcriptional–translational feedback loops, which are mediated by certain clock genes and their protein products, are important to the circadian clock [34]. These loops involve the interplay between genes such as Clock, Bmal1, Per (Period), and Cry (Cryptochrome), which regulate their expression and activity over a 24-h cycle [33]. Members of the basic helix-loop-helix (bHLH)-Period-Arnt-Single-minded (PAS) gene transcription family, Circadian Locomotor Output Cycles Kaput (CLOCK) and Brain and Muscle ARNT-like 1 (BMAL1), are among the positive factors in the major feedback loop. Target genes with E-box cis-regulatory enhancer sequences, such as Period (in mice, PER1, PER2, and PER3) and Cryptochrome (Cry1 and Cry2), are heterodimerized by CLOCK and BMAL1. This results in the transcription of these genes [35,36,37,38]. 

Negative feedback plays a pivotal role in maintaining the oscillatory nature of clock gene expression. This negative feedback loop involves the interaction between the PER (Period) and CRY (Cryptochrome) proteins and the CLOCK: BMAL1 transcriptional complex [36,39,40]. Reverse-erb alpha (Rev-erbα) and Retinoic acid receptor-related orphan receptor alpha (Ror α), two retinoic acid-related orphan nuclear receptors, have their transcription activated by CLOCK: BMAL1 heterodimers, resulting in the induction of another regulatory loop [41,42,43]. Certain DNA sequences (E-box elements) in the promoters of target genes, such as Per (Period) and Cry (Cryptochrome), are bound by the CLOCK-BMAL1 heterodimer [33]. CLOCK-BMAL1 stimulates the transcription of the Per and Cry genes by binding to E-box sites [32]. The messenger RNA (mRNA) produced by the transcription of the Per and Cry genes is translated into the PER and CRY proteins in the cytoplasm [44]. The PER and CRY proteins combine to create complexes in the cytoplasm that grow over time. The nucleus is where the PER/CRY complexes translocate [45]. By suppressing the CLOCK-BMAL1 heterodimer’s transcriptional activity inside the nucleus, the PER and CRY proteins close the feedback loop and regulate their expression. The day–night cycle results in the breakdown of the PER and CRY proteins [46]. The inhibition of CLOCK-BMAL1 is stopped when the levels of PER and CRY drop, enabling the cycle to repeat with a roughly 24-h interval [47]. Numerous post-translational changes, such as phosphorylation and ubiquitination, affect clock genes and the protein products they produce, controlling the genes’ stability, activity, and subcellular localization [48,49]. These adjustments adjust the clock genes’ circadian rhythm control in terms of the timing and accuracy. The expression of downstream target genes involved in the immunological response, hormone production, metabolism, and other physiological functions is regulated by clock genes [50]. Clock-regulated genes express themselves rhythmically, coordinating cellular functions with the daily cycle of light and dark to maximize physiological reactions to external stimuli [51]. 

## 4. The Impact of Irregular Circadian Rhythms on Different Diseases 

As previously highlighted, the circadian rhythm is generated by networks of molecular oscillators in the brain and peripheral tissues that interact with environmental and behavioral cycles to promote the occurrence of sleep during the night [52]. Dysfunction in the 24-h circadian rhythm is a common occurrence in aging adults. However, circadian rhythm disruptions are more severe in people with age-related neurodegenerative diseases, including Alzheimer’s disease-related dementias and Parkinson’s disease. Manifestations differ according to the type and severity of the neurodegenerative disease and, for some patients, occur before the onset of typical clinical symptoms of neurodegeneration [53]. Compared with healthy adults of the same age, patients with moderate to severe Alzheimer’s disease have been considered to have much more severe circadian disruptions, including higher levels of sleep fragmentation, reduced amplitude of circadian rhythmicity, and shifts in both bedtime and wake times to later in the day, known as a “phase delay” [54]. Irregular circadian rhythms are often associated with sleep disorders such as insomnia, delayed sleep phase disorder, and shift work sleep disorder. These conditions can disrupt the quantity and quality of sleep, leading to fatigue, impaired cognitive function, and increased risk of accidents [55]. Damage to the endogenous circadian clock and its synchronization with the round-the-clock social and physical environment are both present in demented individuals. Multiple sleep disorders emerge from this, such as rapid eye movement (REM) sleep disorder, excessive daytime napping, numerous nightly awakenings, delayed initiation of sleep, and night-time roaming [56]. 

It can be difficult to treat sleep and circadian disruptions in adults with dementia. Despite significant progress in comprehending the pathophysiology of the disorders through the circadian rhythm sciences, there are not many empirical data to support successful treatment recommendations (Table 1). 

To develop therapies and ascertain their success in treating patients with dementia, more study is required. Research indicates that the combination of timed light exposure, increased physical and social activities, and sleep hygiene (a planned sleep regimen) can at least partially treat circadian abnormalities. Lately, combining light treatment with a small amount of melatonin has also demonstrated a favorable impact. The multi-component combined modality paradigm can be applied to all dementia patients, even if further research is required to determine the best time and dosage [57]. Abnormal sleep–wake patterns are a basic symptom of mood disorders such as seasonal affective disorder, major depressive disorder and bipolar diseases, and this is indeed one of the primary diagnostic criteria utilized in clinical practice. Furthermore, abrupt disruptions to the sleep–wake cycle might trigger mood disorders. There are now hundreds of research studies that show a link between having a late chronotype (a preference for evening over early activities) and developing a mood disorder. For example, a study of over 2000 persons in the Netherlands discovered a strong connection between a late chronotype and depression/anxiety, even after controlling for sociodemographic, somatic health, and sleep-related characteristics, which was mostly driven by those suffering from depression. Interestingly, having a late time frame was associated with worse health outcomes and an even higher risk of all causes of mortality. This was found in a large study in the United Kingdom (433,268 adults) that compared definite evening types with definite morning types in terms of a wide range of health-related issues. The associations were strongest for psychological disorders and a late chronotype over other health issues [61]. Following that, studies have found that dysregulated circadian rhythms may play a role in the development of metabolic disorders such as metabolic syndrome, diabetes, and obesity. Disrupted sleep patterns can affect the regulation of appetite hormones, leading to overeating and weight gain. Additionally, circadian disruption can impair glucose metabolism and insulin sensitivity, increasing the risk of diabetes [58]. Another increased risk of cardiovascular illness is being linked to abnormal circadian rhythms according to a growing body of data. Hypertension, heart disease, and stroke have been linked to circadian misalignment and disrupted sleep habits (Table 1). This could result from how circadian rhythms affect vascular function, inflammation, and blood pressure regulation [59]. 

Sleep deprivation and disruption are frequent in today’s society. The American Academy of Sleep Medicine and the Sleep Research Society recommend that people need at least 7 h of sleep per day to stay healthy and functional. However, it is uncertain if more than 9 h or less than 7 h of sleep are problematic. Numerous studies have found that a short sleep time is associated with obesity. Longer wakeful times should theoretically result in higher energy expenditure and weight loss, yet short sleep durations typically induce weight gain. It has been proposed that a change in physical activity and a rise in food intake mediate the contradictory link between human obesity and insufficient sleep [62]. 

Increased food intake during sleep disruption can occur through a variety of mechanisms. In human research, sleep deprivation causes alterations in the hormones that stimulate appetite and hunger, such as ghrelin. In comparison to average sleepers, short sleepers typically have lower levels of leptin, higher levels of ghrelin, and higher weights.

Eating at night is nevertheless linked to unintended weight gain when food is readily available overnight. Another study revealed that individuals who slept later had higher BMIs (body mass indexes) and consumed more calories after 8:00 PM. They also consumed fewer fruits and vegetables. Night eating syndrome is linked to a higher BMI and binge eating inclinations. It is defined as not wanting to eat breakfast, consuming ≥50% of daily energy after 7:00 P.M., and experiencing sleep issues ≥ three nights per week [62]. 

The most important aspect is the risk of cancer due to circadian rhythm diseases. Unusual circadian rhythm has been linked to an increased risk of several cancers, according to the data. An increased risk of colon, prostate, and breast cancers has been linked to circadian misalignment and disrupted sleep patterns. This may be because circadian cycles affect hormone control, DNA repair systems, and cell proliferation [60,63].

Circadian rhythms regulate the timing of cell division and DNA repair mechanisms. Disruption to this rhythm can lead to the improper timing of these processes, increasing the likelihood of DNA damage accumulation and mutations, which are key factors in cancer development. From another perspective, circadian rhythms influence the secretion of hormones such as melatonin, cortisol, and growth factors, which play a crucial role in cell growth, proliferation, and apoptosis (cell death) [64].

Alterations in these hormonal rhythms can affect the balance between cell proliferation and cell death, potentially promoting cancer development (Table 1). At the same time, circadian rhythms also regulate the activity of the immune system, including the function of immune cells such as T cells and natural killer cells. The immune system’s capacity to recognize and eradicate cancer cells can be weakened by circadian rhythm disorder [65,66].

The most convincing evidence for circadian changes in skin wound repair was obtained through rhythmic manipulation of fibroblast mobilization. In a study of mouse skin explants collected at different times, wounds healed much faster whether the skin was harvested and wounded at night or during the mouse active phase. Further investigation of the synchronized fibroblast monolayer culture revealed that the increase in healing ability was due to the higher actin assembly efficiency in the invading fibroblasts approaching the wound site. The analysis of the fibroblast proteome indicated that actin lamellipodia assembly was controlled through a cell-intrinsic circadian clock [67]. 

The gut is another system sensitive to circadian regulation of regeneration, with the complete epithelial layer regenerated every five days. Circadian cycles have long been researched to regulate the intestinal epithelial turnover, most commonly in the context of researching the influence of light entrainment and feeding–fasting cycles on the overall proliferative synchronization. Light is the decisive factor in proliferation synchronization; nevertheless, food treatment can impact rhythmicity in the absence of light signals. Recent research has identified multiple circadian regulatory mechanisms in the regeneration of intestinal epithelia, particularly after injury from gastrointestinal (GI) illnesses. Many epidemiological studies have discovered that many diseases are aggravated by circadian rhythm disruption (CRD), such as jet lag, lack of rest, working in shifts, and changes in food and physical activity [67]. 

Another important factor in cancer prevention is avoiding exposure to artificial light at night, such as that from electronic devices or street lights, which can affect the body’s natural circadian rhythms by suppressing the production of melatonin, a hormone involved in regulating sleep–wake cycles that exhibits anticancer properties. Reduced levels of melatonin have been associated with an increased risk of certain types of cancer, including breast and prostate cancer [68,69].

Therefore, it is essential to prioritize healthy sleep habits and minimize disruptions to our circadian rhythms as much as possible. This can include maintaining a consistent sleep schedule, reducing exposure to artificial light before bedtime, and creating a sleep-friendly environment. Additionally, techniques such as light therapy and adjusting meal times can help to realign circadian rhythms when they become disrupted due to factors like jet lag or shift work.

## 5. Cancer Genetics: The Mechanisms

The World Health Organization identified circadian disruption as a probable carcinogen, piquing curiosity about how altering sleep patterns encourages the growth of tumors. In epidemiologic studies, circadian rhythm alterations have been linked to an increased risk of cancer, including cancers of the prostate, breast, colon, liver, pancreas, ovary, and lung. Moreover, a lack of circadian regulation is linked to early cancer patient mortality and inadequate anticancer treatment efficacy [70]. 

Interestingly, sleep, mood disorders, obesity, diabetes, and cancer are all linked to changes in the circadian rhythm caused by eating at night, not getting enough sleep, or chronic jet lag. The expression and activity of several tumor suppressors and oncogenes in both host and tumor tissues are significantly altered by circadian rhythm disturbances caused by genetic and environmental factors. Additionally, circadian disruptions can rearrange the host metabolism and immune systems, instigating an immunosuppressive tumor microenvironment in multiple cancer types [71].

A recent study has demonstrated a direct correlation between apoptosis and the core circadian clock. Circadian factors, depending on the cellular context and clock status, can both promote and restrict apoptosis, as was seen with cell cycle regulation. Cryptochrome (CRY1/2), which is a photoreceptor involved in the circadian clock, and period circadian regulator 1 (PER1) affect the intrinsic apoptotic pathway and the extrinsic tumor necrosis factor alpha-dependent pathway, respectively, in terms of encouraging cell death [72]. 

Furthermore, it has been reported that at least 14 core clock genes form multiple chain feedback loops that provide the molecular clock with its intrinsic circadian rhythmicity. A greater number of studies have shown a strong correlation between the risk of cancer and circadian gene dysfunction caused by single nucleotide polymorphisms (SNPs), deletions, epigenetic modification, and deregulation to the role that clock genes play in the genesis and spread of cancer [73]. 

Of course, attention has been paid to their possible clinical applications as therapeutic targets and predictive biomarkers. The primary circadian pacemaker and the brain’s master clock in mammals is the SCN. It can detect light signals and then transfer them to peripheral clock systems in the liver, muscles, skin, and other tissues. This triggers the transcription factors, which in turn drives the paracrine expression of tissue-specific genes [74]. 

**Figure 2:**
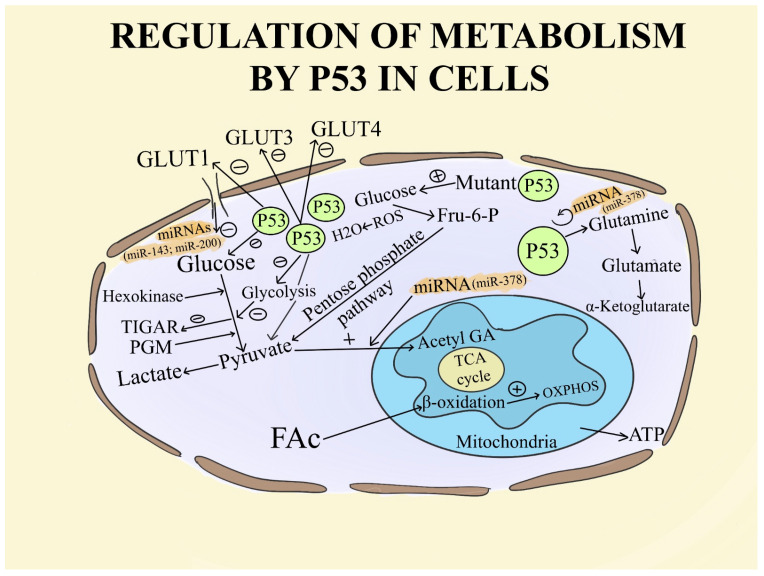
p53 controls the metabolism in cells that are proliferating. p53 suppresses phosphoglycerate mutase (PGM), hexokinase, TP53-induced glycolysis, and apoptosis regulator (TIGAR), and it represses glucose transporters 1 and 4 (GLUT1 and GLUT4). These actions prevent glycolysis from occurring and counteract the Warburg effect, which is observed in many malignancies. The synthesis of glutaminase 2 (GLS2) and cytochrome c oxidase 2 (SCO2) is induced by p53, which increases oxidative phosphorylation. Using IKK/NF-κB signaling, p53 can also control the glycolytic pathway. The Warburg effect is also affected by miRNA dysregulation. miR-143 regulates glycolysis. The miR-200 family controls phosphoglucose isomerase, which is also connected to carcinogenesis. GLUT1 mRNA is a direct target of miR-378a, which limits carcinogenesis and suppresses glucose metabolism in PCa cells. miR-378 influences the TCA cycle in breast cancer. (FAc), fatty acid; TCA, tricarboxylic acid cycle; (−), inhibition; (+), stimulation.

For example, three homologous proteins are encoded by the mammalian period genes: Period regulation 1, 2, and 3. Period Circadian Regulator 2 plays a crucial role in limiting the growth of cells by modulating the expression of multiple genes that are downstream, including Cyclin B1 (CCNB1), which is a protein involved in mitosis, Cyclic D1, which is a protein-coding gene (CCND1), and the TP53 gene that provides instructions for making a protein called tumor protein TP53. It is the primary target of selection pressures in tissues exposed to carcinogens or oncogenic changes due to the suppression proliferation and regulates the survival of stressed cells. Consequently, a selection mechanism inherent to the normal course of cancer could be responsible for the clonal proliferation of cells harboring TP53 mutations [75]. As an essential component of facilitating DNA repair, p53 stops the cell cycle, allowing the repair system adequate time to stabilize the genome. Furthermore, p53 plays several roles that directly affect the functioning of several DNA-repair mechanisms [76]. In the majority of cell types, most data related to p53-associated metabolic activities support the hypothesis that p53 prefers oxidative phosphorylation (OXPHOS) over glycolysis, even though in specialized cells like pancreatic β-cells or hepatocytes, p53 enhances glycolysis and inhibits respiration [77]. According to this theory, p53 deficiency plays a role in cancer cells’ metabolic reprogramming toward a more glycolytic profile. To facilitate glucose absorption, p53 either inhibits the transcription of SLC2A1/4, which encodes the glucose transporters GLUT1/4, or limits IKK–NF-κB activation, which results in a reduction in GLUT3 expression [77,78]. Additionally, p53 suppresses glycolysis by controlling the transcription of other genes that either directly or indirectly affect glycolysis, such as TIGAR, RRAD, PFKFB3/4, and the gene SLC16A1 that encodes the monocarboxylate transporter 1 (MCT1) [79]. The involvement of p53 in this metabolic pathway also includes post-transcriptional mechanisms, which include the downregulation of the glycolytic enzyme phosphoglycerate mutase (PGM) and the regulation of miR-34a, a microRNA that targets multiple glycolytic enzymes [80,81]. Simultaneously, OXPHOS is promoted by p53 through multiple complementary pathways. First, by downregulating pyruvate dehydrogenase (PDH) kinase (PDK2), a negative regulator of the PDH complex (PDC) that transforms pyruvate into Acetyl Coenzyme A (AcCoA) to maintain the tri-carboxylic acid (TCA) cycle, p53 promotes pyruvate oxidation in mitochondria [82]. Restoring p53 function has been demonstrated to raise the α-Ketoglutarate (αKG) levels in pancreatic cancer models, demonstrating the significance of p53 in supplying glucose-derived pyruvate to the TCA cycle. Cancer cells’ epigenome is modified and driven toward a more malignant state by p53 loss, which also changes the activity of αKG-dependent chromatin-changing enzymes by altering the αKG/succinate ratio [83] (Figure 2).

**Figure 3:**
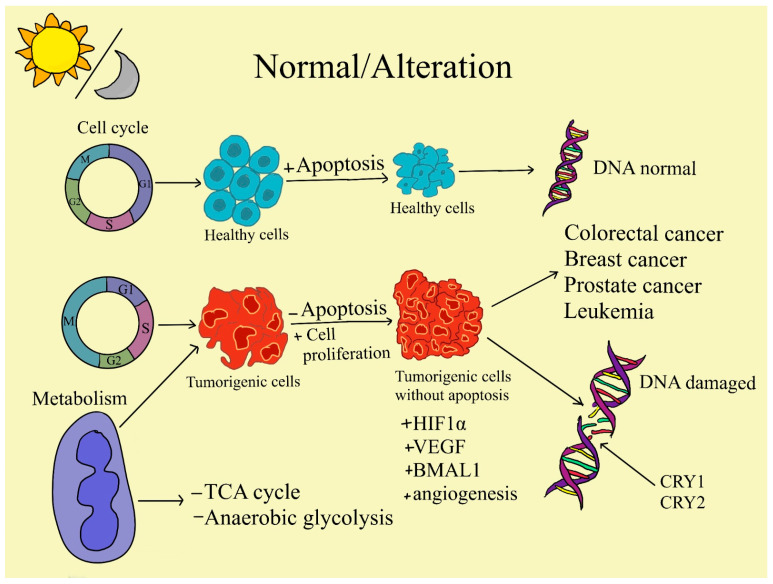
The development of cancer characteristics has been attributed to changes in the circadian rhythm. Circadian rhythms and the cell cycle: DNA replication and the cell cycle present a particular circadian pattern. Circadian rhythms are seen in the expression of DNA replication and cell cycle regulators. Furthermore, different cell cycle genes are modulated by circadian cycle genes. This way, three concepts are tightly regulated: DNA damage response, repair, and circadian rhythms. Cryptochrome 1/2 (CRY1/2). Also, numerous metabolic functions, including the tricarboxylic cycle (TCA) and glucose, are changed by an adequate circadian rhythm. In addition, due to the modification of the malignant medium, vascular endothelial growth factor (VEGF), hypoxia-inducible factor 1-alpha (HIF1α) is highly expressed.

Because PER1 and PER2 are involved in the DNA damage response pathways, abnormal expressions of them may result in cancer. BMAL1 is widely linked to aging, cardiovascular disease, immunological disorders, and cancer. Several investigations have found that BMAL1 regulates the cell cycle and proliferation. CRYs are flavoproteins that respond to blue light and may be found in both plants and mammals. SCN has a high CRY1 expression level, and mice with removed SCN exhibit enhanced tumor development. CRY2 has a unique role in controlling DNA damage repair and genomic stability [73] (Figure 3).

Crucially, the interaction between the circadian clock system and metabolism—which causes metabolic dysrhythmia and circadian rhythm disorder in cancer cells—is mediated by oncogenic factors. One of the primary metabolic regulators that propels cancer is oncogenic MYC. It functions as a transcription factor by binding to the genome’s E-box sites, which are the same as the binding sites of the heterodimeric transcription factor CLOCK-BMAL1 [73].

Throughout tumorigenesis, the transitions between different stages of the cell cycle (G1, S, G2, and M) and different checkpoints are changed. Through the control of cell cycle regulators, circadian rhythm genes are involved in the regulation of the DNA replication program and the rhythmic response to DNA damage. Numerous investigations have demonstrated that the cell cycle checkpoints between the G1/S and G2/M transitions are regulated by the circadian mechanism. Consequently, in cancer, circadian modification may coexist with disruption of the cell cycle. Disruptions to the circadian rhythm in the environment seem to enhance the establishment of distant metastases. These factors can produce inflammatory cytokines and neurotransmitters, activate macrophages, decrease immunological responses, and cause angiogenesis [84].

One important means of cell survival in nutrient-limited environments is autophagy. Animals exhibit diurnal oscillations of autophagy in sync with their circadian cycle because of short-term fasts that induce autophagy. The circadian clock, which is found in nearly all mammalian cell types that have been examined to date, controls the expression of several genes at different times of the day, gating cell functions like proliferation, glycolysis, and food intake. It is unclear at this time whether autophagy in mammalian cells is directly regulated by the circadian clock or if autophagy may contribute to the cycling of mammalian cell clocks. However, the connection between autophagy and circadian cycles is a fascinating topic for further research and has implications for a number of human disorders, including aging, neurodegeneration, and cancer [85]. 

## 6. Circadian Rhythmicity 

Living organisms exhibit rhythmic variations in their behavior and metabolism every 24 h, which serve to anticipate environmental changes. These fluctuations are governed by a sophisticated molecular mechanism known as the circadian clock, which orchestrates the expression of numerous genes to maintain proper bodily function [86]. Additionally, circadian clock genes have been found to regulate autophagy, and disruption of these genes can lead to dysregulated autophagy and an increased cancer risk [87]. 

In particular, it has been demonstrated that overexpression of CLOCK enhances the expression of genes related to angiogenesis, including vascular endothelial growth factor (VEGF), hypoxia-inducible factor 1-alpha, (HIF1α), and basic helix-loop-helix (BMAL1), which is a tumor suppressor (Figure 2). Therefore, by triggering the transcription of RAB27A, a crucial molecule involved in exosome secretion, BMAL1 also seems to contribute to the promotion of metastasis in colorectal cancer [88]. It is important to mention that many types of solid tumors are characterized by hypoxia, a condition whereby cancer cells proliferate quickly to create massive solid tumor masses, which limit and compress the blood arteries surrounding the tumor cells. The central tumor areas frequently receive inadequate oxygenation due to the malfunctioning of these aberrant blood vessels [89]. In this hypoxic area, tumor cells start to adjust to the low level of oxygen by switching on several survival mechanisms. The most well-known mechanism used by hypoxia cells in this hostile milieu is the activation of the HIF-1 transcription factor [90]. Activated HIF-1 plays a crucial role in the adaptive responses of the tumor cells to changes in oxygen through transcriptional activation of over 100 downstream genes, which regulate vital biological processes required for tumor survival and progression. Examples include genes involved in glucose metabolism, cell proliferation, migration, and angiogenesis [91]. Through the overexpression of glucose transporters (GLUTs), which increase glucose import into tumor cells, and the activation of enzymes involved in the glycolysis pathway, HIF-1 mediates this metabolic conversion [92]. An additional example is the HIF-1 transcriptional activation of multiple pro-angiogenic factors, including vascular endothelial growth factor (VEGF). This process promotes the formation of new blood vessels, which supply tumor cells with the oxygen necessary for their growth [93]. Furthermore, by transcriptionally activating neoplastic growth factors, including transforming growth factor beta3 (TGF-β3), epidermal growth factor (EGF), and others, HIF-1 facilitates tumor migration into more distant and more oxygenated tissues [94]

Also, endogenous (~22nt) RNAs, known as miRNAs, can regulate a wide range of biological activities [95]. miRNAs (micro) are implicated in energy metabolism, specifically in the metabolism of fats and carbohydrates as well as the production of amino acids, according to considerable data [96]. Malignant cells may potentially alter their metabolism in order to prevent apoptosis and to encourage the growth and survival of their cells. The most well-studied metabolic phenotype of cancer cells is the Warburg effect, which is caused by miRNA dysregulation and increases glycolysis. Hexokinase 2 (HK2), for example, phosphorylates glucose to produce glucose 6-phosphate (G6P), which provides glucose to the glycolytic process. This is one way that miR-143 regulates glycolysis [97]. Furthermore, it has been demonstrated that the miR-200 family, which consists of miR-200a, miR-200b, and miR-200c, regulates phosphoglucose isomerase, which is also connected to carcinogenesis [98]. Also, it was discovered that GLUT1 mRNA is a direct target of miR-378a, which limits carcinogenesis and suppresses glucose metabolism in PCa cells through numerous pathways. According to this, inhibiting GLUT1 reduces glycolysis, which results in cell death [99]. Likewise, miR-378 influences the TCA cycle in breast cancer [100]. Nevertheless, an increasing amount of data indicate that miRNAs play a significant role in the metabolic control of immune cells in cancer. The phosphoinositide 3-kinase (PI3K)/protein kinase B (AKT) network is a signaling system that controls glucose metabolism by decreasing phosphatase and tensin homolog (PTEN) activities. miRNAs might exert an effect on this pathway, which is located in the inflammatory zone (FIZZ) of M2 macrophages and can upregulate the expression of IL-4, IL-10, transforming growth factor-beta (TGF-β), and arginase [101,102] (Figure 2 and Figure 3).

Studies have demonstrated the role that circadian clock gene dysregulation plays in the development of cancer and other illnesses. The prognosis may perhaps be improved by restoring the circadian rhythms, as disrupting them speeds up the spread of tumors. When compared to normal breast tissues, sporadic and familial breast tumors exhibit lower expression levels of the PER1 and PER2 genes. When comparing sporadic and known forms of breast cancer, the expression levels of the PER1 gene are lower, indicating that the hereditary form of the illness may be caused by a possible disruption to the circadian rhythm. Breast cancer cells may survive if the promoters of the PER1 and CRY1 genes are methylated since this will prevent these genes from being expressed and will interfere with the circadian rhythm of cells [73].

Aging, a family history of the condition, and race are the only known risk factors for prostate cancer. Perhaps a new risk factor for prostate cancer is circadian disturbance. The findings of population-based case-control research support the hypothesis that genetic differences in circadian genes are linked to the development of prostate cancer. In a normal prostate, there are diurnal oscillations in the expression of clock genes and the androgen receptor. PER1 suppresses the androgen receptor’s transcriptional activity, and it appears that prostate cancer is partly caused by the clock gene’s downregulation [103].

Also, numerous cancers, including prostate, breast, colon, liver, pancreatic, ovarian, and lung cancers, have been closely linked to animal models of genetic disruption of CC genes. The expression of the CLOCK protein in tumor cells is significantly decreased in Wilms tumors, a rare kind of kidney cancer that mostly affects children. This suggests that the circadian clock molecular axis may be disturbed in dedifferentiation-mediated embryonal tumors [104]. 

Moreover, previous studies have indicated that people in modern society have sustained a sub-health lifestyle change, including excessive calorie intake at midnight or continuous caloric intake over the 24 h throughout the day, which mimics aspects of shift work and potentially promotes the prostate and breast cancer risk. Impressively, an important element of the circadian clock is the rest/activity rhythm due to its large influence on the treatment of metastatic colorectal cancer patients, who have a poor quality of life with unpredictable intervals of rest/activity [13].

Nevertheless, circadian rhythms are formed by fluctuations in clock gene expression over 24 h. Almost every type of cell activity, including those involved in energy metabolism, cell division, proliferation and apoptosis, ion channels, and signal transduction, has a significant circadian rhythm, according to a comparison of transcripts in both normal and tumor cells from different organs and tissues at different time points and assayed using DNA microarray technology. Circadian rhythms not only synchronize and coordinate sophisticated physiological processes but also allow a system to reset in response to environmental stimulus, resulting in better adaptability to the environment. Another notable fact is that clock genes influence around 50% to more than 80% of the genes in the mammalian genome. These genes, known as clock-controlled genes (CCGs), include the oncogene c-Myc (regulator of cellular metabolism and proliferation) and the tumor suppressor gene p53. CCGs encode a variety of protein products and incorporate cellular rhythmic information. Among them are E-box regulatory elements. Circadian clock genes can have a direct or indirect role in tumor growth by influencing the expression of downstream CCGs involved in cell cycle control, DNA damage repair, cell proliferation and apoptosis, and tumor immunity [13] (Figure 2 and Figure 3).

In another recent study, it was shown that the circadian rhythm transcription factors BMAL1 and CLOCK were demonstrated to be essential for the formation of leukemia stem cells in acute myeloid leukemia (AML), and disruption of circadian pathway components might cause anti-leukemic effects [105]. This investigation used shRNA silencing of BMAL1 and CLOCK, or pharmacological suppression of BMAL1 transcription, to disturb the integrity of the circadian rhythms in both murine and human AML cells, resulting in myeloid differentiation and impaired cell cycle progression. This study identified a unique pro-tumorigenic function of circadian clock genes in AML [105,106] (Figure 2).

However, earlier investigations have demonstrated that the core circadian clock genes are more likely to behave as tumor suppressors in leukemias, where the downregulated expression of the majority of the genes was detected [106]. As SUMOylation, a process in which SUMO proteins are covalently attached to a specific lysine of BMAL1, has been shown to control the circadian clock, and the deregulated methylation status of circadian clock genes has also been demonstrated in various types of cancers, the mechanisms of transcriptional and epigenetic regulation are considered critical for a better understanding of the circadian clock regulation and deregulation during leukemogenesis [106,107]. 

In conclusion, the circadian clock genes and cell cycle work together to maintain a healthy cell function and the malfunction of the gene can lead to tumor growth and can have a big impact on cell homeostasis. The fundamental linkages between circadian rhythms and carcinogenesis have sparked interest in regulating these cycles to avoid malignant transformation, produce more effective medicines or innovative adjuvant methods, and, ultimately, improve cancer patients’ treatment outcomes. 

## 7. The Circadian Clock’s Molecular Pathways in the Development of Cancer 

Specifically, changes in circadian rhythms caused by genetics and environmental factors significantly affect the expression and function of several suppressor genes and cancer genes in the host tissues and the tumor, favoring the development and proliferation of cancer cells [108]. An immunosuppressive tumor microenvironment can be promoted in several cancer types by circadian disturbances that rewire the host metabolism and immune systems. Thus, circadian rhythms are becoming a priority for cancer prevention and treatment in light of their roles in the development and propagation of cancer [109,110]. 

Mammals have their repressors, Period (PER) and Cryptochrome (CRY), whose transcription is cyclically driven by the basic helix-loop-helix ARNT-like 1 (BMAL1) and circadian locomotor output cycles kaput (CLOCK) transcriptional activator complex. The retinoic acid receptor-related orphan receptor (ROR) α/β activator proteins and the REV-ERBα/β repressor preserve the periodic expression of BMAL1 in the second loop, which is complementary to the core oscillator [44]. The molecular clockwork regulates temporal programs via multiple clock output genes and core regulatory loops. It is mediated by various types of epigenetic and post-translational regulation that involve different kinases and phosphatases, ubiquitin-proteasome pathway components, nuclear-cytoplasmic transporters, noncoding RNAs, and chromatin remodelers. The brain and peripheral organ systems maintain this molecular clockwork, forming a body-wide circadian network [111,112].

Clock genes regulate key components of the cell cycle machinery. For instance, the circadian clock modulates the expression and activity of cyclins, cyclin-dependent kinases (CDKs), and checkpoint proteins such as p53. Disruption to circadian rhythms can lead to dysregulation of cell cycle progression, increasing the risk of genomic instability and tumorigenesis [113]. 

Clock genes play a role in coordinating the DNA damage response pathway. Components of the DNA repair machinery, including ATM (ataxia telangiectasia mutated) and CHK2 (checkpoint kinase 2), exhibit circadian oscillations. Disruption to the circadian clock can impair DNA repair processes, resulting in the accumulation of mutations and genomic instability, which are hallmarks of cancer [114]. 

Circadian rhythms influence apoptotic pathways by regulating the expression of pro-apoptotic and anti-apoptotic factors. Clock genes modulate the activity of apoptotic proteins such as the Bcl-2 family members and caspases. Dysregulation of the circadian clock can disrupt the balance between cell survival and apoptosis, contributing to tumor cell evasion of programmed cell death [115,116]. 

Understanding the molecular details of how clock genes interact with cancer pathways provides insights into the underlying mechanisms linking circadian disruption to cancer development and progression. Targeting these pathways may offer novel therapeutic strategies for cancer treatment by restoring circadian rhythms and preventing tumor growth and metastasis [117]. 

As mentioned above, many physiological, pharmacological, molecular, and human behaviors correspond to circadian rhythms [118,119,120]. One example of this is cortisol’s diurnal variation. Another is the pineal gland, which is the primary source of melatonin (N-acetyl-5-methoxytryp tamine). This indoleamine hormone is synthesized from serotonin via the tryptophan-serotonin pathway. The amount of melatonin that is produced varies based on signals from the brain’s circadian regions. It has an impact on pituitary and adrenal hormones, including cortisol, immune system regulation, sleep facilitation, and circadian rhythm regulation [121]. Exposure to light throughout the night, particularly in this period, disturbs the circadian system, which has been linked to cancer development [122]. Melatonin production is likewise suppressed and circadian genes are dysregulated. Cancer-related pathways are modulated when melatonin binds to its receptor, MT-1. One way to understand night-shift work is as an indirect indicator of exposure to night-time light. In the retina-neuroanatomical system, cryptochromes, which are molecular circadian photoreceptors in animals, exhibit varying regional expression patterns. They operate as chromophore cofactors for the electromagnetic signal that is delivered to the circadian molecular clock through the absorption of light by pterin and flavin adenine dinucleotide. They share structural similarities with the enzyme photolyase, which repairs DNA [123]. In contrast to visual photoreceptors based on opsin, cryptochrome circadian regulator 1 (CRY1) and cryptochrome circadian regulator 2 (CRY2) express differently in the retina. Axons from some ganglion cells in the retina go through the retinohypothalamic tract, which emerges from the optic chiasm’s dorsal surface and connects to the SCN. Through crosstalk interaction mediated by vasoactive intestinal polypeptide (VIP) and other neurotransmitters, its neurons are intricately linked and synchronized as an organ [74,124,125]. 

The link between circadian disruptions and cancer etiology has also been supported by the results of multiple investigations using animal models exposed to forced circadian desynchrony regimens. It has been demonstrated that the circadian clock can be altered by SCN ablation or experimental chronic jetlag (CJL), which involves an 8-h increase in the cycle of light and darkness once every 2 days and markedly accelerates the growth rates of transplanted tumors (pancreatic adenocarcinoma and Glasgow osteosarcoma (GSC)) in a mouse model study [126]. Thus, cancer cells may represent an excellent subject for treatment since they tend to be more susceptible than healthy cells to a disruption of clock genes in terms of proliferation [127,128]. Most CRY and REV-ERB agonists effectively prevent the growth of primary GSCs, which are a useful model system for patient variability. Therefore, it would be noteworthy to investigate these agonists in a larger cohort of patient-derived systems in the future. This way, further examination of their subtype specificity and the potential impact of genetic changes and intra-tumor heterogeneity would be better analyzed.

Meal timing is just one aspect of circadian disturbance and its connection to carcinogenesis. It is not limited to light exposure at night. Further evidence that interfering with the biological clock may contribute to the appearance of cancer can be found in a large cohort investigation with individuals from France, which found a correlation between the chance of developing breast or prostate cancer and the late intake of the last meal [129]. A significant amount of time between the previous meal and sleep was similarly linked to a decreased risk of prostate and breast cancer according to another study [130]. It is noteworthy that the majority of research on environmental circadian disturbance and cancer focuses on prostate and breast cancer, suggesting a potential hormonal connection. 

Considering the mentioned studies, it can be concluded that there is a close connection between the circadian clock’s molecular pathways and the development of cancer disease, confirmed by the advancement of this pathology when the molecular clock components change.

## 8. Growth and Multiplication of Cancer Cells Are Regulated throughout the Circadian Clock

Cells are the main components of tissues and organs in humans. They multiply by replicating, and after that, they divide into new cells [131]. In malign tumors, the fundamental characteristic of cancer cells is their capacity to proliferate in an uncontrolled manner, continuously [132]. Also, cancer cells manage to spread throughout the whole human body. Not only the lymphatic system but also the endocrine system plays an important role in the dissemination of cancer cells. Additionally, the lymphatic system transmits information to the cells through hormones. Thus, equally important is the immune system, which defends the organism [131].

**Figure 4:**
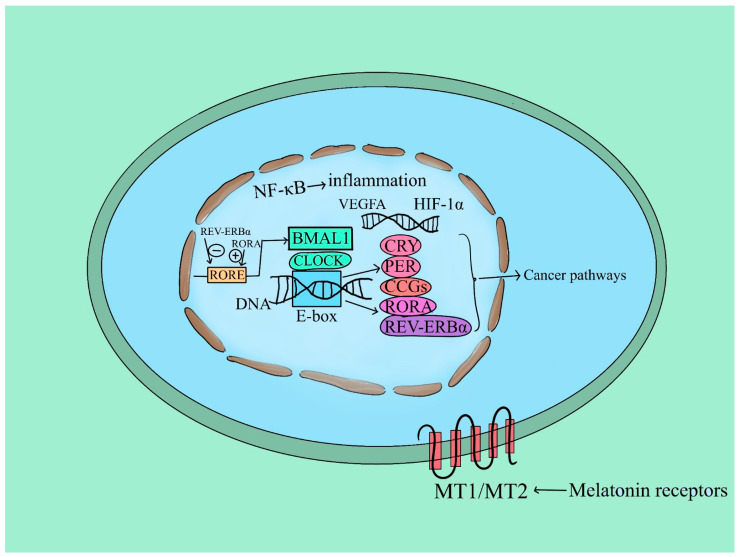
Disrupted circadian rhythm and molecular pathways in the development of cancer. By blocking NF-κB and HIF1α from translocating to the nucleus, melatonin inhibits pathways related to survival and inflammation via binding to the MT1 and MT2 receptors. To activate the transcription of clock-controlled genes (CCGs), RORα, REV-ERBα, CRY (1-2), PER (1-3), and BMAL1 and CLOCK heterodimerize and bind to the E-box. BMAL and CLOCK heterodimer inhibition by CRY and PER creates the main negative feedback loop. BMAL1’s transcription is inhibited by REV-ERBα, while RORα is activated in the secondary feedback loop.

A variety of physiological and behavioral functions in response to the 24-h environmental fluctuations that occur on Earth are regulated by the SCN, which is the area known to be the main stimulator in the mammalian circadian clock [133]. In this sense, CLOCK and BMAL1 are core clock genes, which regulate the expression of numerous target genes in a circadian manner [134]. However, in gene expression patterns and related functions, CLOCK and BMAL1, the circadian transcription factors, are the primary drivers of circadian oscillations, as they are present in nearly every cell of the body [133]. Furthermore, these genes may have a tumor-suppressive role in humans and rodents [72]. Even so, increased cancer susceptibility is also located in clock genes rs117104877, rs2290035, rs2278749, and rs969485 in BMAL1 and rs3749474 and rs11943456 in CLOCK [72]. While many cancer cell types continue to exhibit oscillations of the circadian clock, it was shown that deregulation of the key clock genes PER1, PER2, PER3, CRY1, CRY2, BMAL1, and CLOCK occurs in certain human malignancies. Period (PER) and Crypto-chrome (CRY) are proteins that are referred to as negative regulators of the circadian clock because they can suppress the transcriptional activation capacity of CLOCK and BMAL [135] (Figure 4).

For example, glioma, hepatocellular carcinoma, head and neck squamous cell carcinoma, myeloid leukemia, colorectal, pancreatic, gastric, oral, breast, and non-small-cell lung malignancies all have changed expression levels of PER1, PER2, and PER3. Changes in clock gene expression have also been observed in urological malignancies, including tumors of the prostate, bladder, and kidney. The majority of the clock and clock-associated genes are expressed differently in distinct cancer types according to genomic research. A study conducted by Kinouchi et al. [136] showed that the PER1 gene was found to be suppressed in glioma, breast and prostate cancers, and PER2 in leukemia, lung, and stomach cancer. Moreover, PER3 was found to be suppressed in colorectal cancer. Regardless, an increase in PER3 expression was observed in acute lymphoid leukemia. Also, tumor growth can be suppressed and apoptosis can also be induced by overexpression of PER1 in cell lines [136]. Equally important, the CRY family genes were also found to have complex roles in cancer types. In colorectal cancer cells, increased CRY1 was associated with tumor advancement [137]. On the other hand, breast cancer is correlated with better survival in high expression levels of CRY2 [138]. In contrast, in liver cancer, a low level of CRY2 was directly proportional to a shorter survival [139]. Papillary and follicular thyroid carcinoma were also associated with lower levels of CRY2 [140].

In a study conducted on mice by Yang et al. [141], the involvement of PER1 in the growth and proliferation of breast cancer cells has been investigated. They concluded that the circadian clock, which has two daily peaks and troughs, controls the growth rate of breast cancer. These peaks and troughs are correlated with the daily expression patterns of clock-controlled genes that oversee cell proliferation. At specific times of the day, PER1 has a tumor suppressor function that diminishes the proliferation of cancer cells and tumor growth. The circadian amplitude of the two daily tumor growth peaks is improved by a decrease in PER1 expression (Figure 3). Thus, PER1 promotes the growth of cancer cells in vitro and tumors in vivo. Likewise, the clock gene PER2 in mammalians is responsible for inhibiting the formation of tumors and the proliferation of cancer cells, both in vivo and in vitro [141].

Furthermore, another study has proved that clock genes were downregulated in tumors and B16 cells, which are melanoma cell lines of murine origin. However, therapies that induced circadian rhythmicity, like heat shock, forskolin, and dexamethasone, stimulated the expression of rhythmic clock and cell cycle genes, leading to a decrease in S-phase cells and an increase in G1-phase cells [142]. As a result, there was a slowdown in the growth of tumors in vivo and B16 proliferation in vitro. Similar changes were reported in HCT-116, isolated human colon cancer cells from an adult male. Even so, neither an increase in apoptosis nor an improvement in immune cell recruitment to the tumor was the cause of dexamethasone’s effects. Dexamethasone’s effects on tumor growth and cell cycle events were inhibited in B16 tumors by knocking down the key clock gene BMAL1. The tumor-intrinsic circadian clock mediates the effects of dexamethasone on the cell cycle and tumor growth. Therefore, a strategy for the control of cancer progression might be the enhanced circadian clock function [142].

The degradation of oncoproteins like E2F, a transcription factor that regulates the expression of genes in cell proliferation, TLK2 (tousled-like kinase 2), cell cycle arrest, and apoptosis in cancer is promoted by the increased expression or activity components of the circadian clock (CLOCK, BMAL1) [71,143]. The circadian clock also controls DNA repair, EMT (epithelial–mesenchymal transition), metabolism, and inflammation.

Circadian rhythms play a crucial role in regulating DNA repair processes, ensuring their optimal function in response to environmental and endogenous cues. Research has shown that various components of the DNA repair machinery, such as nucleotide excision repair (NER), base excision repair (BER), and double-strand break repair (DSB), exhibit rhythmic patterns of activity, peaking at different times of the day [144].

For instance, studies have demonstrated that the expression and activity of key DNA repair genes, including those involved in NER and BER, are under the control of circadian clock genes. These clock genes regulate the transcription of DNA repair genes, thereby modulating the efficiency of the DNA repair processes in a time-of-day-dependent manner [145].

Moreover, disruption to circadian rhythms, either experimentally or due to factors such as shift work or jet lag, can impair the DNA repair capacity and increase the susceptibility to DNA damage-induced mutations. This dysregulation of DNA repair has been implicated in various health conditions, including cancer, neurodegenerative diseases, and aging.

Understanding the intricate relationship between circadian rhythms and DNA repair systems is crucial for elucidating the mechanisms underlying disease development and for developing novel therapeutic strategies. Harnessing the rhythmic nature of DNA repair processes may hold promise for optimizing the timing of cancer treatments, reducing the risk of genomic instability, and improving overall health outcomes [146].

Correspondingly, it prevents the transformation and the spread of tumors by maintaining the temporal homeostasis of cells. As a result, numerous biological functions regulate the clock mechanism, causing interconnections throughout the pathways. A complex correlation between the circadian clock genes and cell proliferation is suggested by studies, but further research is needed for a better understanding of regulating tumorigenesis using the circadian clock genes [147].

Recent studies have revealed that the deregulation of GSK3b (glycogen synthase kinase 3 beta) promotes tumor cell survival, proliferation, invasion, and resistance to chemo- and radiation therapy in humans by inhibiting p53 (tumor suppressor protein) and RB (retinoblastoma) tumor suppressors, inducing intracellular NF-kB (nuclear factor kappa-light-chain-enhancer of activated B cells) signaling, Cyclin D1 (promotes G1/S cell cycle progression) overexpression, and local chronic inflammation [148]. Tumor cell survival is sustained by GSK3b in many cancer types, such as pancreatic [149], non-small-cell lung carcinoma [150], renal cell carcinoma [151], and chronic lymphocytic leukemia B cells [152]. The activity of GSK3b exhibits robust circadian rhythm in both SCN and peripheral tissues, suggesting that GSK3b may also indirectly target PER2 in the mammalian molecular clock.

Combining the previously presented findings, it appears that the molecular clock rhythmically paces the essential physiological pathways that promote cell proliferation and prevent tumor growth, associating cell proliferation with mammalian daily physiology [153].

## 9. Cancer and the Roles of Circadian Clock Components

Activities that interfere with the endogenous balance through external circadian signals have become more prevalent as the world has become more industrialized. This alteration in lifestyle has been associated with a higher risk of all types of illnesses, including cancer, in humans [154]. Many studies have shown that the loss of circadian homeostasis in the energy balance, immunological response, and aging is closely linked to cancer development in vivo [147]. These effects are reinforced by cellular processes critical for tumor suppression, such as metabolism, senescence, cell division, and DNA damage response [153].

The circadian clock plays a pivotal role in modulating cellular responses to DNA damage, encompassing processes such as repair, checkpoints, and apoptosis [155]. Cells activate surveillance mechanisms termed DNA damage checkpoints, which intricately regulate the progression of the cell cycle by arresting or decelerating its course. These checkpoint signaling pathways within mammalian cells are well-characterized, notably the ATR → Chk1(checkpoint 1) pathway, elicited by UV-mimetic agents and compounds impeding replication fork progression, and the ATM → Chk2 (checkpoints 2) pathway, primarily induced by double-strand breaks resulting from ionizing radiation (IR) and radiomimetic agents [156]. Upon recognition by the ATM and ATR sensor kinases, facilitated by accessory proteins, the damage signal is transduced to Chk1 and Chk2 kinases via mediators. Effector proteins such as tumor protein p53 [157], Cell Division Cycle 25 (Cdc25) [158], and Cell Division Cycle 45 (Cdc45) undergo phosphorylation by these signal-transducing kinases. Consequently, the phosphorylation of these proteins leads to the inhibition of two crucial kinases, Cell Division Cycle 2 kinase (Cdc2), and Cyclin-Dependent Kinase 2 (Cdk2), inducing cell cycle arrest. Importantly, the DNA damage checkpoint response not only arrests the cell cycle but also instigates specific DNA repair pathways aimed at reinstating replication forks and rectifying double-strand breaks [159].

Circadian clocks, regulated by both genetic and epigenetic factors, exhibit self-sustaining oscillations synchronized to environmental cues [160]. Recent advancements have shed light on the intricate interplay between the circadian clock and epigenetic plasticity. This mutual correlation is evident in histone modifications, noncoding Ribonucleic Acid (RNA) production—primarily microRNA (miRNA)—and genomic DNA methylation, all of which are influenced by the circadian clock [161]. Conversely, these epigenetic mechanisms exert indirect and direct effects on clock output genes, modulating the cyclic transcription and translation of core circadian genes (Figure 3). Significant insights have emerged concerning the interconnection of the circadian clock, epigenetics, and cancer, particularly in breast, colorectal, and hematologic malignancies. Aberrant methylation of circadian gene promoters and dysregulated miRNA production have been implicated in altering circadian gene expression and the 24-h expression oscillation tempo [162].

The immune system represents a complex network of physiological processes geared toward protecting the body against non-self-substances, encompassing cancer cells and pathogens such as bacteria, viruses, and parasites. Circadian clocks are ubiquitously present in the majority of cell types, inclusive of immune system cells [163].

Additionally, daily oscillations characterize the synthesis and release of key immune mediators, including cytokines, chemokines, and cytolytic factors, alongside the temporal gating of immune responses via pattern recognition receptors. Consequently, disruptions to circadian rhythms lead to perturbations in immunological responses, underscoring the critical role of circadian regulation in maintaining immune homeostasis [65].

The body’s myriad cells are equipped with circadian clocks, pivotal for synchronizing physiological functions and behavioral patterns with diurnal rhythms. Within the hypothalamic–pituitary–adrenal (HPA) endocrine axis, glucocorticoids—produced by the adrenal cortex—regulate responses to acute and chronic stress [164]. Crucially, the circadian rhythm of glucocorticoid secretion, orchestrated downstream of the SCN, coordinates peripheral clocks and rhythms. The research underscores glucocorticoids’ potent anti-inflammatory properties and their influence on immune cells, suggesting an intimate interplay between stress and circadian systems in immune regulation [66]. Cell proliferation is one of the biological processes governed by circadian rhythm, and it frequently exhibits asynchrony between normal and malignant tissues [165]. This asynchrony serves as one of the theoretical cornerstones for cancer chronotherapy and emphasizes the role of the circadian clock in tumor suppression in vivo.

New therapeutic targets may result from research into the processes by which the circadian clock regulates biological activities, including cell proliferation [166]. Chronotherapy in cancer treatment involves timing anticancer medications based on circadian rhythms to enhance effectiveness and minimize harm to healthy tissues [167,168]. While conventional chemotherapy often falls short of achieving full remission in many cases, clinical trials conducted across multiple centers have demonstrated the therapeutic efficacy of chronotherapy [169]. Specifically, chronotherapeutic regimens utilizing oxaliplatin have proven safe and effective in treating metastatic colorectal cancer, leading to unprecedented long-term survival rates. This approach presents promising avenues for expanding cancer treatment options and optimizing the development of new supportive or anticancer medications [167].

As shown, a strong relationship exists between the circadian clock components and modulating cellular responses to DNA damage, including repair, checkpoints, and apoptosis. Thus, it can be stated that circadian clock components specifically affect the development and proliferation of cancer disease (Figure 3).

## 10. How Do Clock Components Affect Particular Types of Cancer?

During a normal circadian cycle, the immune system displays biphasic shift, which maintains the equilibrium of T-helper1 (Th1) cell-derived cytokines: IL-2, IL-12, INFN-γ, and T-helper2 (Th2) cell-derived cytokines: IL-4, IL-10 [12,13]. In the first hours of sleep, the Th1 activity is enhanced and accompanied by the moderate promotion of interferon (IFN)-γ/IL-4-producing Th cells, while the activity of Th2 dominates in the last part of sleep or even before waking up [13,170]. Once this rhythm is interrupted, cytokines begin to be excessively produced, resulting in disturbances to the immune system. This fact inevitably leads to chronic inflammation and tissue damage [13,170]. Different cytokines, like TNF-α, TGF-β, IL-10, and especially IL-1β and IL-6, were demonstrated to be involved in the crosstalk complex between cancer initiation/progression and sleep–wake cycles [13,171,172]. Notable, IL-1β, which gains access through passive diffusion, was shown to be a key mediator affecting rhythmical behavior [13,173].

Interestingly, numerous studies have proven that this type of rhythm disturbance, associated with low levels of melatonin (a tumorigenesis initiation suppressor and inhibitor of cancer cell lines) [174,175], which are most often present among shift workers, over time leads to a much higher risk of developing breast cancer among women and prostate cancer among men [147,176]. Regarding breast cancer, circadian genes like NPAS2, CLOCK, RORA, RORB, and PER3 are responsible for the formation and activation of this type of cancer [177,178].

Pepłońska et al. [177] conducted a cross-sectional study about this kind of disease and how is it linked to the chronotype (morning and evening chronotype). Following this study, they concluded that prolonged (more than 15 years) night shifts could be associated with a higher level of estradiol in the morning chronotype in the case of postmenopausal women [179,180]. Hurley et al. also found that individuals with a definite evening chronotype were more susceptible to developing mammalian cancer than those with a clear morning chronotype [180,181]. Estradiol represents a major source of estrogenic activity. This is mainly converted in estrone through the 17β-hydroxysteroid dehydrogenase. Estrone is subsequently metabolized by two major pathways [182,183]: through the 2-hydroxylase pathway to the catechol estrogens and through the 16α-hydroxylase pathway to 16α-hydroxy-estrone, and then estriol 16α-hydroxy-estrone subsequently binds covalently to proteins and nucleotides following the Heyns rearrangement [184]. This type of binding was postulated to result in a prolonged estrogenic effect [185,186]. The catechol estrogens also bind weakly to the estrogen receptor and are considered to be weak estrogens, especially in breast cancer. Formation of these estrogens depends on a cytochrome P450 mono-oxygenase (present in most tissues). They can be formed in estrogen-dependent tissues such as the breast [187].

As is well known, cancer cells exhibit impaired expressions of core clock genes. That is how deregulations of cell proliferation, metabolism, induction, invasion, and migration are produced [188,189,190]. Regarding prostate cancer (PCan), various clock genes, such as ARNTL, CLOCK, BMAL1, Ck1ε, CRY1, CRY2, Npas2, and PER1-3, are found to be involved in this type of carcinoma [153,190,191]. The polymorphisms of these genes advance the development of this kind of tumoral cell [192]. A study conducted by Chu et al. [191] showed that low levels of the PER3 gene, and different variants of the CRY2 gene, are directly related to the expression of PCan [191]. In this case, the melatonin receptor located in the SNC is the one that regulates PER, CRY, BMAL1, and RORα gene expression to prevent the appearance of tumoral cells [192] (Table 2).

Therefore, melatonin is the one that enhances PER2 and CLOCK and decreases BMAL1 in this type of carcinoma [193]. Androgens, especially testosterone, are synthesized in the testicles. However certain fragments of androgen hormones are produced in the reticular zone of adrenal glands. This type of hormone binds to the androgen receptors (ARs) to perform their normal function. Different mutations of ARs determine the aggressiveness of PCan [194,195]. In this case, melatonin mediates the nuclear exclusion of ARs and even blocks their activity. This determines AR mislocalization, from which the antitumor effect results [196]. Also, in normal values, melatonin decreases follicle-stimulating hormone (FSH) and luteinizing hormone (LH) secretion. This effect decreases hypothalamic–pituitary–gonadal (HPG) axis-mediated gonadal steroidogenesis [197]. Thus, suppression of steroidogenesis in males can inhibit the growth of PCan [198]. Moreover, cancer cell progression is linked to metabolic reprogramming (Table 2). The prostate is a glandular tissue that secretes citrate in prostatic fluid. Normal cells use glucose for citrate synthesis but never for citrate-oriented metabolism and oxidative phosphorylation. Instead, abnormal cells need this type of oxidative process to proliferate [199,200].

Tumoral cells express GLUT1 (a transmembrane protein responsible for the facilitated diffusion of glucose across a membrane) as an optimal source of glucose [201]. Thus, melatonin inhibits metabolic alteration to prevent the survival of cancer cells. It competitively binds with GLUT1 and suppresses its expression [200,202].

In addition, angiogenesis represents the crucial step in the progression of PCan [203]. The rate of angiogenesis depends on vascular endothelial growth factor (VEGF) expression. The cancerous cells cause high levels of VGEF, or a form of it, VGEF-A, to potentiate angiogenesis [201,204]. Regulators of VGEF expression are represented by both the androgenic-AR complex and hypoxia-inducible factor 1α (HIF-1α). Accelerated growth of tumor cells causes hypoxic conditions. This ultimately causes HIF-1α, which will result in the expression of hundreds of oncogenes [205]. In this case, melatonin has the role of an HIF-1α stabilization inhibitor and HIF-1α-induced gene expression restrictor [188] (Figure 2). Any interruption to and disturbance of the circadian rhythm of those mentioned earlier in this paper negatively influences the immune system activity, secretion, levels, and ability of melatonin to act and inhibit tumor cells. The tumor probability and extent are higher in individuals with chronic circadian rhythm disturbance. Moreover, it raises the possibility and intensity of comorbidities that obstruct cancer treatment [206].

## 11. The Relationship between the Circadian Clock and Cancer Treatment

Chronotherapy represents the timing of drug delivery at the appropriate phase of the circadian rhythm to achieve its optimal efficacy. The reasoning behind this method is based on three key points. Primarily, the effectiveness of a drug can fluctuate throughout the day, contingent on its mode of action [178,207]. Second, pharmacokinetics and the metabolism of agents may also vary with the circadian phase [178,208]. Finally, the toxic activity of agents may also vary with the time of day [178,209]. Generally, existing evidence does not substantiate the assertion that chronochemotherapy is universally advantageous for treating all types of cancer, and it is not a widely adopted practice. Nevertheless, chronochemotherapy protocols that are meticulously designed could potentially enhance the treatment strategies for specific cancers. To fully harness the potential of this approach in cancer treatment, there is a need for a chronochemotherapy regimen that is based on mechanisms. This regimen should align the optimal circadian timing with the specific drug target, availability, metabolism, and toxicity. Platinum-based drugs like cisplatin, which eradicate cancer cells by inflicting DNA damage, are used in the treatment of numerous solid-tissue tumors. The global and gene-specific repair processes have been thoroughly studied in mice and human cell lines [178,210,211].

Following more murine-based studies, the rate of repair is low in the morning and at a maximum in the evening [212], and, as predicted, mice treated in the evening exhibited higher repair than those treated in the morning, though this difference was abolished by the *Per1/2-/-* mutation [213]. However, it is important to mention that murine species are nocturnal species, while humans are diurnal. For practical clinical use, besides the circadian repair pattern of the genome in normal tissues, it would be necessary to have detailed repair maps of specific types of cancer. These would help in determining the best timing to selectively eliminate cancer cells. Studies examining the circadian gene expression patterns in particular cancers are still rare. The findings have ranged from cancer tissue being arrhythmic [214,215], to others indicating that it has a rhythm but is out of sync with normal tissue [216], and to still others demonstrating that it has a rhythm and is in sync with normal tissue [217].

Establishing the relationship between the circadian phase and amplitude in cancer and normal tissues is crucial for the creation of truly mechanism-based chronotherapy regimens. A significant hurdle in achieving this is the development of noninvasive techniques to evaluate the circadian parameters of cancers. Metabolic processes and the transcription and expression of different genes control their function. Statistical analysis of clinical trials, both in vivo and in vitro experiments, have demonstrated the significant research value of biological CLOCK in treating tumors and controlling the antitumor efficacy of chemotherapy and radiation therapy [218].

Furthermore, the transcription–translation feedback loop (TTFL) is organisms’ biochemical foundation of circadian rhythms. The TTFL controls the primary molecular circadian clock mechanism. The TTFL is often found in animals’ anterior hypothalamus, SCN, and peripheral tissues. It is stated that CLOCK’s interaction with small molecules (CLK8) reduces CLOCK’s interaction with BMAL1 and obstructs CLOCK’s translocation into the nucleus. The nucleus’s decreased CLOCK contributes to the TTFL’s more stable negative arm and strengthens the circadian clock’s function. Thus, CLK8 improves aging, emotional disorders, and the efficacy of cancer treatment by influencing the activities of CLOCK and BMAL1 [33]. Nobiletin, a naturally occurring polymethoxy flavonoid compound, is another small molecule that may potentially improve the CLOCK effect by raising the PER2 level and stabilizing the negative arm of the TTFL as a result. The small molecules are the main players and possible targets for developing new treatments for illnesses like cancer, which are connected to disruptions to the circadian rhythm. They may also improve the activities of CLOCK and BMAL1 [219].

**Figure 5:**
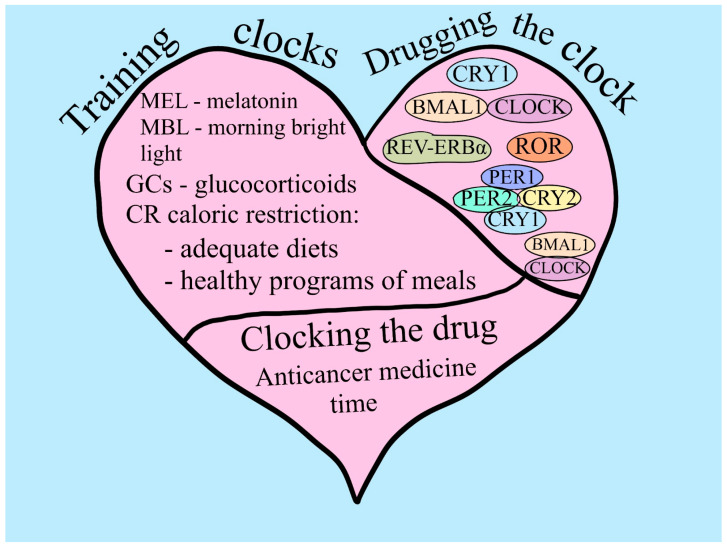
Chronobiological methods for preventing and treating cancer and its associated disorders. Different diseases can be the result of disruptions to the circadian rhythm. Thus, a deeper understanding of the relationship between the two may prevent cancer. In this way, three categories of chronotherapeutic therapies are important. (1) Clocking the drugs: maximizing drug timing to enhance efficacy and minimize negative side effects; circadian rhythms significantly impact the pharmacokinetics and pharmacodynamics of medication responses. The effectiveness and bioavailability of the medications are influenced by rhythmic changes, which might range from drug absorption to target receptor expression. (2) Administering the clock: using small-molecule drugs that specifically target a circadian clock. (3) Training the clock: appropriate strategies to improve or sustain a healthy circadian periodicity in feeding–fasting, sleep–wake, or light-dark cycles.

Although a disturbance to the circadian rhythm can lead to certain diseases, a greater knowledge of how these two relate can be used to improve cancer prevention. The circadian rhythms have an impact on pharmacodynamics and pharmacokinetics, just as they do on other biological activities [220]. Pharmacokinetics establishes the ideal drug concentration required to generate a toxic-to-effective ratio. Consequently, drug absorption, distribution, metabolism, and excretion—also referred to as ADME processes—constitute the four distinct phases of pharmacokinetic processes. They can be utilized for chronotoxicity and chronoefficacy studies because they exhibit circadian rhythmicity and express various drug-metabolizing enzymes and transporters [221]. Understanding how circadian rhythms could be utilized to enhance the treatment of various diseases is becoming more and more popular in modern times [222]. In a recent article, chronotherapeutic treatments were classified into three kinds: (1) training the clock: adequate strategies to improve or sustain a healthy circadian periodicity in feeding–fasting, sleep–wake, or light–dark cycles; (2) administering the clock: utilizing small-molecule drugs that specifically target a circadian clock; and (3) clocking the drugs: optimizing drug timing to improve efficacy and reduce adverse side effects [223]. Consequently, the investigation of circadian rhythms and the modality that influences a drug’s reaction is known as chronotherapy or clinical chronopharmacology. The aim is to enhance the drug’s positive effects while reducing the possible potential negative side effects for the patient. Pharmacological or therapeutic interventions are thus applied at a particular and ideal time according to the patient’s circadian rhythm, which is known as chronotherapy [224] (Figure 5).

These ideas are not mutually exclusive and have been used in combination to treat cancer. For example, training the clock can be used to improve weakened or disordered rhythms, enhancing the effects of timed medications, or drugging the clock. With these ideas in mind, the next sections present contemporary chronobiological approaches to cancer treatment [225].

Also, tumor cells often include tumor-associated antigens (TAAs) as well as tumor-specific antigens (TSAs). These antigens are released into the plasma upon tumor cell death and then collected by dendritic cells (DCs) and macrophages. DCs, macrophages, and B cells are strong antigen-presenting cells (APCs) in the human body. When triggered by antigens or inflammatory cytokines, including Interleukin 1 beta (IL-1β), Tumor Necrosis Factor (TNF-α), and Transforming Growth Factor-β (TGF-β), immature DCs can develop into mature DCs that express major histocompatibility complex molecules (MHC II) on their surfaces [226]. Simultaneously, the expression of costimulatory and adhesion molecules on their surfaces increases considerably. The antigens are then collected from the peripheral organs to prime and activate the T lymphocytes. Furthermore, DCs can produce IL-12, which causes T cells in tumor microenvironments (TMEs) to develop into T helper (Th) cells, promoting the elimination and growth suppression of tumor cells. Effective molecular clocks in DCs, such as CLOCK, PER, and BMAL-1, exhibit daily oscillations and suggest that the host DCs are under circadian control [227].

In the same manner, circadian clock components can direct cancer antigen-specific T and natural killer (NK) cells to destroy cancer cells. Immune evasion represents a negative prognostic marker and predictor of immune checkpoint blockade response in several malignancies. Effective immunotherapeutic techniques have been developed to treat cancer. BMAL1 expression in metastatic melanomas is responsible for T cell activation/differentiation signals, as well as T cell fatigue markers. Advanced analysis of the functional impact of circadian clock genes revealed that the circadian clock-enriched pathways are supplemented in many immune-related pathways, including programmed death-ligand (PD-L1) expression and programmed cell death protein 1 (PD-1) checkpoint pathway in cancer, T cell receptor signaling pathway, and TNF signaling pathway; this confirms that the circadian clock widely regulates tumor immunity [228]. For this reason, several anticancer treatments are anticipated to lower medication toxicity while also improving the tumor response rate and duration. Interferon-β (IFN-β), a pleiotropic cytokine involved in immune system modulation, showed a stronger anticancer impact in tumor-bearing mice during the early light phase compared to the early dark phase. In phase I/II clinical investigation, IL-2 chronotherapy was used to treat metastatic renal cell cancer and showed modest toxicity, feasibility in a standard care unit, and activity [229]. These findings might be useful in developing properly rationalized pharmacological therapies for modulating circadian clock components, which may be changed in tumors [230].

Another way of treating cancer is by using chronotherapy, which is the administration of drugs at the proper period of the circadian cycle to ensure maximum effectiveness. Although the use of chronotherapy for specific pathologic disorders such as asthma, hypertension, and cardiovascular disease has resulted in considerable advances in medication regimen development, it has not been used for cancer treatment. However, all treatment techniques are based on the preferential death of cancer cells over normal cells. Apoptosis is a primary mechanism of drug-induced cell death. Analyses of the effect of clock disruption on the cellular response to DNA damage revealed that the circadian clock influences apoptosis against specific genetic backgrounds, indicating that this effect should be addressed in chronotherapy regimens [231].

The first discovery that the clock influences apoptosis stemmed from research into the effect of clock disruption on carcinogenesis. To investigate the influence of CRY mutations on carcinogenesis, CRY1/2-/- mutations were paired with a p53-null mutation, which is a standard technique for determining the carcinogenicity of weakly penetrant tumorigenic genes. The p53-/- mice, as predicted, developed lymphoma and lymphosarcoma and lived an average of 5.5 months. Contrary to expectations, the p53-/-CRY1/2-/- mice showed a lower age-adjusted cancer incidence and lived 1.5 times longer than the p53-/- mice. This discovery led to the hypothesis that against the p53-/-CRY1/2-/- background, cells become more sensitive to DNA-damaging agents. These findings are briefly summarized here to highlight the potential for enhanced sensitivity to apoptosis as a variable in cancer treatment [155].

On the other hand, the emerging basic functions of core clock components in cancer biology have prompted the development and investigation of small compounds that directly target clock proteins. Fortunately, current clock-targeting small medicines have shown specific tumor growth suppression in glioblastoma and other cancer types. Given the diverse involvement of the core clock components in cancer management, it is advantageous for future research to create cancer-specific techniques to enhance traditional first-line regimens. A deeper knowledge of clock–cancer interactions will help to develop blood or tissue-based biomarkers for molecular classification of cancer patients who may respond differently to clock-targeting therapy [232].

The functional spectrum of the circadian clock has to be expanded to guide pharmacological therapies through more mechanistic research in various cancer processes and pathways, supported by clinical and omics data obtained from cancer patients.

## 12. Clocking Medication to Treat Cancer

The debate keeps on going about the specifics of the clock–cancer relationship. Circadian rhythm disruption is observed in a multitude of tumors [233,234], and the absence of circadian control is linked to the initiation and advancement of cancer. Genetic and environmental changes in circadian rhythms exacerbate the vulnerability of cancer pathogenesis [89,139]. On the other hand, a few malignancies contain a functioning circadian clock that causes daily fluctuations in gene expression [142,235], but less is understood about how these clocks affect the development or management of tumors. However, it can be more successful by administering chemotherapy under the host’s circadian rhythms (Figure 4). Moreover, it is yet unknown where and what kind of circadian regulation is needed [213,236]. Generally, the lack of experimental models and mechanistic evidence for circadian-modulated drug activity has limited the scope of timed chemotherapy treatment for cancer (chronochemotherapy) [237].

In humans, inadequate sleep and circadian misalignment have been identified as risk factors for obesity and associated metabolic diseases. Large cohort studies have shown that obesity increases the incidence of several main types of cancer, including endometrial, postmenopausal breast, and colorectal cancers, owing to a variety of causes. These processes include higher levels of estrogen, insulin, insulin-like growth factor 1, and leptin, decreased secretion of the antiproliferative adipocyte-derived adiponectin, and the persistence of chronic inflammation. Training the clock with TRE/TRF (ten-hour time-restricted eating/two weeks of early time-restricted feeding) has been demonstrated to be successful in preventing obesity and metabolic illnesses, as well as inhibiting tumor development and attenuating metastasis in postmenopausal obesity-driven breast cancer and obesity-enhanced tumor metastasis models. Circadian regulation governs the diurnal oscillation of GC (glucocorticoid) secretion via the hypothalamic–pituitary–adrenal axis (HPA). GC serves as a primary circadian and stress signal. Chronic circadian disturbances, either by misaligned light exposure or food consumption, can significantly change daily levels of GC and stress-induced GC responses [238].

According to some research, two different kinds of circadian rhythm-related cancer treatments are on the market: ones that target the regulators of circadian core clock genes and those that can directly alter the activity of circadian core genes. The second category of drugs is not unique to clock components; instead, they address other target proteins, such as those that phosphorylate or degrade clock components. Among these are substances that target the F-box protein Fbxw7, casein kinase CK1ε, and (CK) 1δ [239].

It is well recognized that the two CK1 family members, CK1δ and CK1ε, are crucial for controlling the circadian clock system and that pharmacological suppression of these members affects circadian rhythms in vivo [239,240]. Anticancer therapy is interested in targeting CK1δ and ε, as they are highly expressed in several tumor types such as leukemia, breast, pancreas, and ovarian cancer [241]. It is interesting to note that human renal cell carcinoma (RCC) lines’ ability to proliferate is impacted by newly created, strong CK2 inhibitors [242] (Figure 4).

Also, REV-ERBα is ubiquitinated and degraded more readily when Fbxw7 is present [243]. In vitro and xenograft models show that tumor development is inhibited when Fbxw7’s nuclear maintenance is forced via the use of specific inhibitors of nuclear export (SINEs) [244]. Oridonin, a plant extract, contains a chemical that requires Fbxw7 to have some anticancer effects. Nevertheless, it is not evident if the drugs that target Fbxw7 and CKs have anticancer effects that are primarily mediated through their circadian clock targets [245].

A conserved circadian clock in cancer cells has been shown to play a crucial role in modulating responses to anticancer drugs, according to research using cell-based pharmacological techniques to evaluate the impact of daytime (chronopharmacology). Also, it has been shown that medications that target HSP90 work in a time-dependent way because some rhythmically produced HSP90 isoforms modify the cell cycle. It is generally accepted that oncogenic Myc or Ras overexpression causes clock processes in cancer cells to be disrupted or dysregulated, at least in part [234]. Nevertheless, despite their strong predisposition for tumorigenesis and metastasis, certain cancer cells, like B16 murine melanoma models and U2OS human bone tumors, can be made to exhibit circadian rhythms using dex [142,246]. A study has demonstrated that U2OS cells, which are generated from osteosarcomas, can also respond temporally to anticancer drugs [89]. Patient-derived cancer stem cells (CSCs) from glioblastoma or acute myeloid leukemia have strong circadian rhythms irrespective of the elevated MYC expression levels, which is consistent with both experimental tumor models [105,127]. Many isoforms of the heat shock protein 90 (HSP90)-targeted drugs may regulate their antitumor properties; hence, it is important to determine which isoform is relevant for time-of-day specificity. The effects that different Hsp90 isoforms have on cellular differentiation and development, as well as gene expression and subcellular localization, are important details [247]. Mammalian Hsp90AA1 and Hsp90AB1 possess a similar structure and cytosolic expression. However, Hsp90AA1 is constitutively generated, mostly for cellular repair, whereas Hsp90AB1 is activated by external signals to promote oncogenic signaling responses and cell cycle progression [248]. In the same way as Hsp90AA1, Hsp90B1, an endoplasmic reticulum-localized subtype of Hsp90, interacts with a variety of pro-survival and mitogenic proteins to facilitate the growth and spread of cancer [249].

Also, the use of clock medications on IL-17A-expressing T cells in vivo to enhance chimeric antigen receptor (CAR)-T cell treatment is a viable tactic. T lymphocytes of type 17 are regulated by the master transcription factor RORγt. RORγt-primed T cells in mice protect against cancer when tumor cells are reintroduced into the animal, and the RORγt agonist supplementation during ex vivo expansion improves the anticancer efficacy of Th17 cells modified with CAR [250,251]. In vivo, RORγ agonists can also operate as monotherapy. Crucially, the treatment of RORγ agonists has anticancer attributes due to the activation of several tumor suppressive pathways, such as increasing Th17 cell activity and obstructing Treg-driven immunosuppression [250]. T cell proliferation and survival are improved by the administration of RORγ agonists, which additionally enhances the production of GM-CSF, IL-17A, and the costimulatory receptors CD137 and CD226. Furthermore, RORγ agonists impair Treg activity by downregulating the expression of CD39 and CD73, and they also attenuate the expression of coinhibitory receptors PD-1 and TIGIT [250]. An improved form of the RORγ agonist is currently undergoing clinical studies as an independent drug (clinical investigation NCT0292862) or in combination with pembrolizumab (anti-PD-1; clinical study NCT03396497) for the treatment of patients with established, recurrent, or resistant solid cancers. No significant adverse events were noted in the Phase I study [147].

Thus, pharmacological regulation of proteins in the circadian machinery may provide a fresh chance to improve other targeted treatment efficacy by acting on important cancer-related pathways. To this end, the relationship between the circadian clock and the immune system may provide a valuable chance to improve the immunotherapy efficacy and potentially overcome the resistance mechanisms. The molecular clock’s main components, including BMAL1, CLOCK, and REV-ERBα, regulate immune cell formation, activity, and trafficking, altering cancer immune surveillance. Additionally, it has been demonstrated that the core BMAL1::CLOCK transcription apparatus is crucial for maintaining stemness in at least two distinct cancer types. These findings indicate that utilizing clock-targeting small molecules to lower BMAL1::CLOCK activity and inhibit CSCs (central serous chorioretinopathy) may be a successful tactic for improving cellular sensitivity to non-targeted conventional therapy [252]. Many different aspects of illness also exhibit circadian cycles. Throughout a 24-h day, the strength of the indications and symptoms varies regularly. This also applies to how responsive human beings are to medicine. This is where the idea of chronotherapy enters the picture: administering treatment at the best time of day to maximize therapeutic outcomes (Figure 5).

## 13. Conclusions

This review has highlighted the crucial significance of preserving a circadian rhythm in homeostasis because it regulates different physiological processes over a day. These cycles influence hormone release, physiological functions, sleep–wake cycles, and other bodily processes. By timing medical operations to align with these circadian cycles, chronotherapy aims to reduce side effects and maximize therapeutic efficacy. Thus, this paper explores the intricate relationship between circadian rhythms and chronotherapy, specifically in the context of cancer, and discusses future research targets in this fascinating field.

(1) Cancer chronotherapy is a promising field that aims to improve efficacy and reduce negative effects by synchronizing cancer therapies with the body’s circadian cycles [253,254].
(2) The circadian clock and human health are tightly related, with a phenotypic impact that includes illnesses such as cancer disease. In this way, disruptions to the circadian clock could damage both tumor molecular clocks and circadian mechanisms, increasing the cancer risk and cell progression by altering hormone levels, immune function, and metabolic pathways
(3) Additionally, the aforementioned results suggest that the molecular clock regularly controls the essential physiological pathways that promote cell proliferation and prevent tumor growth, connecting cell proliferation with mammalian daily physiology. In this sense, it has been demonstrated that an important connection exists between the components of the circadian clock and control of cellular responses to DNA damage, such as repair, checkpoints, and apoptosis. In this way, the elements of the circadian clock particularly influence the onset and spread of cancer.
(4) It is becoming clear that clock genes and circadian regulators may be useful therapeutic targets in the fight against cancer. Using pharmaceutical methods (targeting clock proteins) or lifestyle interventions (improving sleep patterns) to modify the circadian clock may provide innovative ways to prevent or treat cancer. Through additional mechanistic studies in multiple cancer processes and pathways, supported by clinical and omics data collected from cancer patients, the functional spectrum of the circadian clock needs to be expanded to influence pharmaceutical therapy. A question also remains regarding how responsive people are to medical treatment. Thereby, chronotherapy comes into play for treating patients during the most effective time of day to optimize their recovery.
(5) However, let us not forget that each patient is genetically distinct and therefore has their own circadian rhythm. From a financial point of view, adopting these strategies is a true challenge in the long term to administer treatment according to the circadian characteristics of each patient.
(6) Moreover, it is critical to draw attention to healthy sleeping practices and reduce any interruptions to our circadian cycles to the greatest extent possible. This can involve creating a sleep-friendly environment, keeping to a regular sleep schedule, and minimizing artificial light exposure prior to bedtime. Furthermore, methods like light therapy and timing mealtimes can assist in re-establishing circadian rhythms when they are disturbed by things like jet lag or shift work. From a future perspective, it will be interesting to observe if adopting all three criteria for an optimal rhythm can decrease or even reverse the cancer risk following disturbances to the molecular clock. It is also noteworthy to find out how much the last meal’s composition might affect sleep and, consequently, the circadian rhythm. The consumption of healthy, easily digestible foods, such as those in the Mediterranean diet, is recommended, especially for individuals who work night shifts and cannot skip dinner.
(7) Also, to make progress in this field, scientists must expand their comprehension of the molecular mechanisms that underlie circadian rhythms and how these rhythms impact cellular functions, especially in cancer cells. Important insights may be gained by examining the circadian gene expression patterns in different malignancies and how these patterns differ from those in normal tissues.
(8) Furthermore, discovering techniques for adapting chronotherapy regimens according to each patient’s unique circadian profile—which may be determined by wearable technology or biological biomarkers—will improve precision medicine techniques. It is also essential to research how the time of medication administration affects anticancer drug pharmacokinetics and pharmacodynamics.
(9) Personalized treatment plans may be created by using wearable technology to track patients’ circadian rhythms in real time. Big data analytics and intelligent methods (artificial intelligence) can be used to examine trends in circadian rhythms and forecast the best times to administer treatments.

## Figures and Tables

**Table 1:** Diseases related to circadian rhythm disruptions.

Circadian Disruptions	Diseases	Effects	References
	Alzheimer’s	Neurodegeneration	[52]
	Insomnia, Delayed sleep phase disorder, Shift work sleep disorder	Weariness, Deteriorated cognitive performance, Increased accident risk	[22,54]
	Disruption of regulation of appetite hormones	Overeating, Weight gain	[55]
	Impairing of glucose metabolism and insulin sensitivity	Increased risk of diabetes	[57]
	Hypertension, Heart disease, Stroke	Affects vascular function, inflammation, and blood pressure regulation	[58]
	Cancer	Increased risk of prostate and breast cancers	[59,60]

**Table 2:** Gene expression related to different types of cancer.

Gene	Type of Cancer	Effects	References
PER3, CRY2	Prostate cancer	Expression of PCan	[191]
BMAL1, RORα	Prostate cancer	Prevent the appearance of tumoral cells	[192]
NPAS2, CLOCK, RORA, RORB, PER3	Breast cancer	Expression of breast cancer	[177,178]

## Data Availability

Data are contained within the article.

## References

[1] Gnocchi D., Bruscalupi G. (2017). Circadian rhythms and hormonal homeostasis: Pathophysiological implications. Biology.

[2] Roy P.S., Saikia B. (2016). Cancer and cure: A critical analysis. Indian J. Cancer.

[3] Schwartz S.M. (2024). Epidemiology of cancer. Clin. Chem..

[4] Vaghari-Tabari M., Ferns G.A., Qujeq D., Andevari A.N., Sabahi Z., Moein S. (2021). Signaling, metabolism, and cancer: An important relationship for therapeutic intervention. J. Cell. Physiol..

[5] López-Otín C., Diamandis E.P. (1998). Breast and prostate cancer: An analysis of common epidemiological, genetic, and biochemical features. Endocr. Rev..

[6] Jensen O.M. (2019). Colon cancer epidemiology. Experimental Colon Carcinogenesis.

[7] Zhang J.-M., An J. (2007). Cytokines, inflammation, and pain. Int. Anesthesiol. Clin..

[8] Kartikasari A.E., Huertas C.S., Mitchell A., Plebanski M. (2021). Tumor-induced inflammatory cytokines and the emerging diagnostic devices for cancer detection and prognosis. Front. Oncol..

[9] Al-Abri M.A., Al-Yaarubi S., Said E.A. (2023). Circadian rhythm, sleep, and immune response and the fight against COVID-19. Oman Med. J..

[10] Richards J., Gumz M.L. (2013). Mechanism of the circadian clock in physiology. Am. J. Physiol.-Regul. Integr. Comp. Physiol..

[11] Ruan W., Yuan X., Eltzschig H.K. (2021). Circadian rhythm as a therapeutic target. Nat. Rev. Drug Discov..

[12] Dimitrov S., Lange T., Fehm H.L., Born J. (2004). A regulatory role of prolactin, growth hormone, and corticosteroids for human T-cell production of cytokines. Brain Behav. Immun..

[13] Zhou L., Zhang Z., Nice E., Huang C., Zhang W., Tang Y. (2022). Circadian rhythms and cancers: The intrinsic links and therapeutic potentials. J. Hematol. Oncol..

[14] U.S. Department of Health and Human Services (2023). Circadian Rhythms.

[15] Albrecht U. (2004). The mammalian circadian clock: A network of gene expression. Front. Biosci..

[16] Moore R.Y., Speh J.C., Leak R.K. (2002). Suprachiasmatic nucleus organization. Cell Tissue Res..

[17] Bass J., Takahashi J.S. (2011). Redox redux. Nature.

[18] Morrison S.F., Gebber G.L. (1985). Axonal branching patterns and funicular trajectories of raphespinal sympathoinhibitory neurons. J. Neurophysiol..

[19] Reddy S., Reddy V., Sharma S. (2018). Physiology, circadian rhythm. StatPearls [Internet].

[20] Amaral F., Cipolla-Neto J. (2018). A brief review about melatonin, a pineal hormone. Arch. Endocrinol. Metab..

[21] Cagnacci A., Krauchi K. (1997). Homeostatic versus circadian effects of melatonin on core body temperature in humans. J. Biol. Rhythm..

[22] Brown G.M. (1994). Light, melatonin and the sleep-wake cycle. J. Psychiatry Neurosci..

[23] Antle M.C., Smith V.M., Sterniczuk R., Yamakawa G.R., Rakai B.D. (2009). Physiological responses of the circadian clock to acute light exposure at night. Rev. Endocr. Metab. Disord..

[24] Cajochen C., Kräuchi K., Wirz-Justice A. (2003). Role of melatonin in the regulation of human circadian rhythms and sleep. J. Neuroendocrinol..

[25] Sack R.L., Lewy A.J., Blood M.L., Keith L.D., Nakagawa H. (1992). Circadian rhythm abnormalities in totally blind people: Incidence and clinical significance. J. Clin. Endocrinol. Metab..

[26] Yamanaka Y., Hashimoto S., Masubuchi S., Natsubori A., Nishide S.Y., Honma S., Honma K.I. (2014). Differential regulation of circadian melatonin rhythm and sleep-wake cycle by bright lights and nonphotic time cues in humans. Am. J. Physiol.-Regul. Integr. Comp. Physiol..

[27] Morris C.J., Aeschbach D., Scheer F.A. (2012). Circadian system, sleep and endocrinology. Mol. Cell. Endocrinol..

[28] Kanaley J.A., Weltman J.Y., Pieper K.S., Weltman A., Hartman M.L. (2001). Cortisol and growth hormone responses to exercise at different times of day. J. Clin. Endocrinol. Metab..

[29] Veldhuis J.D., Iranmanesh A., Lizarralde G., Johnson M.L. (1989). Amplitude modulation of a burstlike mode of cortisol secretion subserves the circadian glucocorticoid rhythm. Am. J. Physiol. -Endocrinol. Metab..

[30] Pietrowsky R., Meyrer R., Kern W., Born J., Fehm H.L. (1994). Effects of diurnal sleep on secretion of cortisol, luteinizing hormone, and growth hormone in man. J. Clin. Endocrinol. Metab..

[31] Takahashi J.S. (2015). Molecular components of the circadian clock in mammals. Diabetes Obes. Metab..

[32] Ko C.H., Takahashi J.S. (2006). Molecular components of the mammalian circadian clock. Hum. Mol. Genet..

[33] Partch C.L., Green C.B., Takahashi J.S. (2014). Molecular architecture of the mammalian circadian clock. Trends Cell Biol..

[34] Takahashi J.S. (2004). Finding new clock components: Past and future. J. Biol. Rhythm..

[35] King D.P., Zhao Y., Sangoram A.M., Wilsbacher L.D., Tanaka M., Antoch M.P., Steeves T.D.L., Vitaterna M.H., Kornhauser J.M., Lowrey P.L. (1997). Positional cloning of the mouse circadian clock gene. Cell.

[36] Kume K., Zylka J., Sriram S., Shearman L., Weaver D. (1999). mCRY1 and mCRY2 are essential components of the negative limb of the circadian clock feedback loop. Cell.

[37] Bunger M.K., Wilsbacher L., Moran S., Clendenin C., Radcliffe L. (2000). Mop3 is an essential component of the master circadian pacemaker in mammals. Cell.

[38] Zheng B., Albrecht U., Kaasik K., Sage M., Lu W., Vaishnav S., Li Q., Sun Z.S., Eichele G., Bradley A. (2001). Nonredundant roles of the mPer1 and mPer2 genes in the mammalian circadian clock. Cell.

[39] Lee C., Etchegaray J., Cagampang F., Loudon A., Reppert S. (2001). Posttranslational mechanisms regulate the mammalian circadian clock. Cell.

[40] Sato T.K., Yamada R.G., Ukai H., Baggs J.E., Miraglia L.J., Kobayashi T.J., Welsh D.K., Kay S.A., Ueda H.R., Hogenesch J.B. (2006). Feedback repression is required for mammalian circadian clock function. Nat. Genet..

[41] Preitner N., Damiola F., Lopez-Molina L., Zakany J., Duboule D. (2002). The orphan nuclear receptor REV-ERBα controls circadian transcription within the positive limb of the mammalian circadian oscillator. Cell.

[42] Sato T.K., Panda S., Miraglia L.J., Reyes T.M., Rudic R.D., McNamara P., Naik K.A., FitzGerald G.A., Kay S.A., Hogenesch J.B. (2004). A functional genomics strategy reveals Rora as a component of the mammalian circadian clock. Neuron.

[43] Triqueneaux G., Thenot S., Kakizawa T., Antoch M.P., Safi R., Takahashi J.S., Delaunay F., Laudet V. (2004). The orphan receptor Rev-erbα gene is a target of the circadian clock pacemaker. J. Mol. Endocrinol..

[44] Takahashi J.S. (2017). Transcriptional architecture of the mammalian circadian clock. Nat. Rev. Genet..

[45] Lee Y., Jang A., Francey L., Sehgal A., Hogenesch J. (2015). KPNB1 mediates PER/CRY nuclear translocation and circadian clock function. eLife.

[46] Hirota T., Fukada Y. (2004). Resetting mechanism of central and peripheral circadian clocks in mammals. Zool. Sci..

[47] Zheng B., Larkin D.W., Albrecht U., Sun Z.S., Sage M., Eichele G., Lee C.C., Bradley A. (1999). The mPer2 gene encodes a functional component of the mammalian circadian clock. Nature.

[48] Lee Y., Lee J., Kwon I., Nakajima Y., Ohmiya Y., Son G.H., Lee K.H., Kim K. (2010). Coactivation of the CLOCK–BMAL1 complex by CBP mediates resetting of the circadian clock. J. Cell Sci..

[49] Tamaru T., Hirayama J., Isojima Y., Nagai K., Norioka S., Takamatsu K., Sassone-Corsi P. (2009). CK2α phosphorylates BMAL1 to regulate the mammalian clock. Nat. Struct. Mol. Biol..

[50] Curtis A.M., Bellet M.M., Sassone-Corsi P., O’Neill L.A. (2014). Circadian clock proteins and immunity. Immunity.

[51] Green C.B., Takahashi J.S., Bass J. (2008). The meter of metabolism. Cell.

[52] Meyer N., Harvey A.G., Lockley S.W., Dijk D.J. (2022). Circadian rhythms and disorders of the timing of sleep. Lancet.

[53] Leng Y., Musiek E.S., Hu K., Cappuccio F.P., Yaffe K. (2019). Association between circadian rhythms and neurodegenerative diseases. Lancet Neurol..

[54] Videnovic A., Lazar A.S., Barker R.A., Overeem S. (2014). The clocks that time us—Circadian rhythms in neurodegenerative disorders. Nat. Rev. Neurol..

[55] Rose K.M., Lorenz R. (2010). Sleep disturbances in dementia: What they are and what to do. J. Gerontol. Nurs..

[56] McCurry S.M., Ancoli-Israel S. (2003). Sleep dysfunction in Alzheimer’s disease and other dementias. Curr. Treat. Options Neurol..

[57] Zhou Q.P., Jung L., Richards K.C. (2012). The management of sleep and circadian disturbance in patients with dementia. Curr. Neurol. Neurosci. Rep..

[58] Zimmet P., Alberti K.G.M.M., Stern N., Bilu C., El-Osta A., Einat H., Kronfeld-Schor N. (2019). The Circadian Syndrome: Is the Metabolic Syndrome and much more!. J. Intern. Med..

[59] Takeda N., Maemura K. (2011). Circadian clock and cardiovascular disease. J. Cardiol..

[60] Stevens R.G., Hansen J., Costa G., Haus E., Kauppinen T., Aronson K.J., Castaño-Vinyals G., Davis S., Frings-Dresen M.H.W., Fritschi L. (2011). Considerations of circadian impact for defining ‘shift work’ in cancer studies: IARC Working Group Report. Occup. Environ. Med..

[61] Dollish H.K., Tsyglakova M., McClung C.A. (2024). Circadian rhythms and mood disorders: Time to see the light. Neuron.

[62] Noh J. (2018). The effect of circadian and sleep disruptions on obesity risk. J. Obes. Metab. Syndr..

[63] Li T., Jiang Y., Bai Y., Jiang K., Du G., Chen P., Luo C., Li L., Qiao J., Shen J. (2023). A review for the impacts of circadian disturbance on urological cancers. Sleep Biol. Rhythm..

[64] Chen Z., McKnight S.L. (2007). A conserved DNA damage response pathway responsible for coupling the cell division cycle to the circadian and metabolic cycles. Cell Cycle.

[65] Labrecque N., Cermakian N. (2015). Circadian clocks in the immune system. J. Biol. Rhythm..

[66] Baxter M., Ray D.W. (2020). Circadian rhythms in innate immunity and stress responses. Immunology.

[67] Paatela E., Munson D., Kikyo N. (2019). Circadian regulation in tissue regeneration. Int. J. Mol. Sci..

[68] Cho Y., Ryu S.H., Lee B.R., Kim K.H., Lee E., Choi J. (2015). Effects of artificial light at night on human health: A literature review of observational and experimental studies applied to exposure assessment. Chronobiol. Int..

[69] Tähkämö L., Partonen T., Pesonen A.-K. (2019). Systematic review of light exposure impact on human circadian rhythm. Chronobiol. Int..

[70] de Assis L.V.M., Oster H. (2021). The circadian clock and metabolic homeostasis: Entangled networks. Cell. Mol. Life Sci..

[71] Lee Y. (2021). Roles of circadian clocks in cancer pathogenesis and treatment. Exp. Mol. Med..

[72] Shafi A.A., Knudsen K.E. (2019). Cancer and the circadian clock. Cancer Res..

[73] Liu H., Liu Y., Hai R., Liao W., Luo X. (2023). The role of circadian clocks in cancer: Mechanisms and clinical implications. Genes Dis..

[74] Welsh D.K., Takahashi J.S., Kay S.A. (2010). Suprachiasmatic nucleus: Cell autonomy and network properties. Annu. Rev. Physiol..

[75] Guimaraes D., Hainaut P. (2002). TP53: A key gene in human cancer. Biochimie.

[76] Williams A.B., Schumacher B. (2016). p53 in the DNA-damage-repair process. Cold Spring Harb. Perspect. Med..

[77] Schwartzenberg-Bar-Yoseph F., Armoni M., Karnieli E. (2004). The tumor suppressor p53 down-regulates glucose transporters GLUT1 and GLUT4 gene expression. Cancer Res..

[78] Kawauchi K., Araki K., Tobiume K., Tanaka N. (2008). p53 regulates glucose metabolism through an IKK-NF-κB pathway and inhibits cell transformation. Nat. Cell Biol..

[79] Boidot R., Végran F., Meulle A., Le Breton A., Dessy C., Sonveaux P., Lizard-Nacol S., Feron O. (2012). Regulation of monocarboxylate transporter MCT1 expression by p53 mediates inward and outward lactate fluxes in tumors. Cancer Res..

[80] Kim H.R., Roe J.S., Lee J.E., Cho E.J., Youn H.D. (2013). p53 regulates glucose metabolism by miR-34a. Biochem. Biophys. Res. Commun..

[81] Kondoh H., Lleonart M.E., Gil J., Wang J., Degan P., Peters G., Martinez D., Carnero A., Beach D. (2005). Glycolytic enzymes can modulate cellular life span. Cancer Res..

[82] Contractor T., Harris C.R. (2012). p53 negatively regulates transcription of the pyruvate dehydrogenase kinase Pdk2. Cancer Res..

[83] Morris J.P., Yashinskie J.J., Koche R., Chandwani R., Tian S., Chen C.C., Baslan T., Marinkovic Z.S., Sánchez-Rivera F.J., Leach S.D. (2019). α-Ketoglutarate links p53 to cell fate during tumour suppression. Nature.

[84] Ortega-Campos S.M., Verdugo-Sivianes E.M., Amiama-Roig A., Blanco J.R., Carnero A. (2023). Interactions of circadian clock genes with the hallmarks of cancer. Biochim. Biophys. Acta (BBA)-Rev. Cancer.

[85] Sachdeva U.M., Thompson C.B. (2008). Diurnal rhythms of autophagy: Implications for cell biology and human disease. Autophagy.

[86] Amiama-Roig A., Verdugo-Sivianes E.M., Carnero A., Blanco J.R. (2022). Chronotherapy: Circadian rhythms and their influence in cancer therapy. Cancers.

[87] Li H., Li M., Chen K., Li Y., Yang Z., Zhou Z. (2022). The circadian clock gene ARNTL overexpression suppresses oral cancer progression by inducing apoptosis via activating autophagy. Med. Oncol..

[88] Lee Y., Lahens N.F., Zhang S., Bedont J., Field J.M., Sehgal A. (2019). G1/S cell cycle regulators mediate effects of circadian dysregulation on tumor growth and provide targets for timed anticancer treatment. PLoS Biol..

[89] Powis G., Kirkpatrick L. (2004). Hypoxia inducible factor-1α as a cancer drug target. Mol. Cancer Ther..

[90] Semenza G.L. (2002). HIF-1 and tumor progression: Pathophysiology and therapeutics. Trends Mol. Med..

[91] Semenza G.L. (2001). HIF-1 and mechanisms of hypoxia sensing. Curr. Opin. Cell Biol..

[92] Denko N.C. (2008). Hypoxia, HIF1 and glucose metabolism in the solid tumour. Nat. Rev. Cancer.

[93] Conway E.M., Collen D., Carmeliet P. (2001). Molecular mechanisms of blood vessel growth. Cardiovasc. Res..

[94] Laderoute K.R., Calaoagan J.M., Gustafson-Brown C., Knapp A.M., Li G.C., Mendonca H.L., Ryan H.E., Wang Z., Johnson R.S. (2002). The response of c-Jun/AP-1 to chronic hypoxia is hypoxia-inducible factor 1α dependent. Mol. Cell. Biol..

[95] Chen B., Li H., Zeng X., Yang P., Liu X., Zhao X., Liang S. (2012). Roles of microRNA on cancer cell metabolism. J. Transl. Med..

[96] Rottiers V., Näär A.M. (2012). MicroRNAs in metabolism and metabolic disorders. Nat. Rev. Mol. Cell Biol..

[97] Cairns R.A., Harris I.S., Mak T.W. (2011). Regulation of cancer cell metabolism. Nat. Rev. Cancer.

[98] Ahmad A., Aboukameel A., Kong D., Wang Z., Sethi S., Chen W., Sarkar F.H., Raz A. (2011). Phosphoglucose isomerase/autocrine motility factor mediates epithelial-mesenchymal transition regulated by miR-200 in breast cancer cells. Cancer Res..

[99] Cannistraci A., Hascoet P., Ali A., Mundra P., Clarke N.W., Pavet V., Marais R. (2022). MiR-378a inhibits glucose metabolism by suppressing GLUT1 in prostate cancer. Oncogene.

[100] Eichner L.J., Perry M.C., Dufour C.R., Bertos N., Park M., St-Pierre J., Giguère V. (2010). miR-378∗ mediates metabolic shift in breast cancer cells via the PGC-1β/ERRγ transcriptional pathway. Cell Metab..

[101] Lu J., Xie L., Liu C., Zhang Q., Sun S. (2017). PTEN/PI3k/AKT regulates macrophage polarization in emphysematous mice. Scand. J. Immunol..

[102] Wang D., Wang X., Si M., Yang J., Sun S., Wu H., Cui S., Qu X., Yu X. (2020). Exosome-encapsulated miRNAs contribute to CXCL12/CXCR4-induced liver metastasis of colorectal cancer by enhancing M2 polarization of macrophages. Cancer Lett..

[103] Savvidis C., Koutsilieris M. (2012). Circadian rhythm disruption in cancer biology. Mol. Med..

[104] Santoni M., Molina-Cerrillo J., Santoni G., Lam E., Massari F., Mollica V., Giulia Mazzaschi G., Rapoport B., Grande E., Buti S. (2023). Role of clock genes and circadian rhythm in renal cell carcinoma: Recent evidence and therapeutic consequences. Cancers.

[105] Puram R.V., Kowalczyk M.S., de Boer C.G., Schneider R.K., Miller P.G., McConkey M., Ebert B.L. (2016). Core circadian clock genes regulate leukemia stem cells in AML. Cell.

[106] Yang M.-Y., Lin S.-F. (2016). The role of circadian clock genes in leukemia. Transl. Cancer Res..

[107] Cardone L., Hirayama J., Giordano F., Tamaru T., Palvimo J.J., Sassone-Corsi P. (2005). Circadian clock control by SUMOylation of BMAL1. Science.

[108] Papagiannakopoulos T., Bauer M.R., Davidson S.M., Heimann M., Subbaraj L., Bhutkar A., Matthew G., Vander Heiden V.G.M., Jacks T. (2016). Circadian rhythm disruption promotes lung tumorigenesis. Cell Metab..

[109] Aiello I., Fedele M.L.M., Román F., Marpegan L., Caldart C., Chiesa J.J., Golombek D.A., Finkielstein C.V., Paladino N. (2020). Circadian disruption promotes tumor-immune microenvironment remodeling favoring tumor cell proliferation. Sci. Adv..

[110] Hadadi E., Taylor W., Li X.M., Aslan Y., Villote M., Rivière J., Duvallet G., Auriau C., Dulong S., Raymond-Letron I. (2020). Chronic circadian disruption modulates breast cancer stemness and immune microenvironment to drive metastasis in mice. Nat. Commun..

[111] Anafi R.C., Lee Y., Sato T.K., Venkataraman A., Ramanathan C., Kavakli I.H., Hughes M.E., Baggs J.E., Growe J., Liu A.C. (2014). Machine learning helps identify CHRONO as a circadian clock component. PLoS Biol..

[112] Lee Y., Shen Y., Francey L.J., Ramanathan C., Sehgal A., Liu A.C., Hogenesch J.B. (2019). The NRON complex controls circadian clock function through regulated PER and CRY nuclear translocation. Sci. Rep..

[113] Farshadi E., van Der Horst G.T., Chaves I. (2020). Molecular links between the circadian clock and the cell cycle. J. Mol. Biol..

[114] Collis S.J., Boulton S.J. (2007). Emerging links between the biological clock and the DNA damage response. Chromosoma.

[115] Wang Y., Qian R., Sun N., Lu C., Chen Z., Hua L. (2015). Circadian gene hClock enhances proliferation and inhibits apoptosis of human colorectal carcinoma cells in vitro and in vivo. Mol. Med. Rep..

[116] Zhu L., Wang Q., Hu Y., Wang F. (2019). The circadian gene Per1 plays an important role in radiation-induced apoptosis and DNA damage in glioma. Asian Pac. J. Cancer Prev. APJCP.

[117] Fagiani F., Di Marino D., Romagnoli A., Travelli C., Voltan D., Di Cesare Mannelli L., Marco Racchi M., Govoni S., Lanni C. (2022). Molecular regulations of circadian rhythm and implications for physiology and diseases. Signal Transduct. Target. Ther..

[118] Panda S., Antoch M.P., Miller B.H., Su A.I., Schook A.B., Straume M., Takahashi J.S., Hogenesch J.B. (2002). Coordinated transcription of key pathways in the mouse by the circadian clock. Cell.

[119] Storch K.F., Lipan O., Leykin I., Viswanathan N., Davis F.C., Wong W.H., Weitz C.J. (2002). Extensive and divergent circadian gene expression in liver and heart. Nature.

[120] Kita Y., Shiozawa M., Jin W., Majewski R.R., Besharse J.C., Greene A.S., Jacob H.J. (2002). Implications of circadian gene expression in kidney, liver and the effects of fasting on pharmacogenomic studies. Pharmacogenetics Genom..

[121] Malhotra S., Sawhney G., Pandhi P. (2004). The therapeutic potential of melatonin: A review of the science. Medscape Gen. Med..

[122] Stevens R.G., Blask D.E., Brainard G.C., Hansen J., Lockley S.W., Provencio I., Rea M.S., Reinlib L. (2007). Meeting report: The role of environmental lighting and circadian disruption in cancer and other diseases. Environ. Health Perspect..

[123] Sancar A. (2000). Cryptochrome: The second photoactive pigment in the eye and its role in circadian photoreception. Annu. Rev. Biochem..

[124] Aton S.J., Colwell C.S., Harmar A.J., Waschek J., Herzog E.D. (2005). Vasoactive intestinal polypeptide mediates circadian rhythmicity and synchrony in mammalian clock neurons. Nat. Neurosci..

[125] Liu A.C., Welsh D.K., Ko C.H., Tran H.G., Zhang E.E., Priest A.A., Buhr E.D., Singer O., Meeker K., Verma I.M. (2007). Intercellular coupling confers robustness against mutations in the SCN circadian clock network. Cell.

[126] Filipski E., Lévi F. (2009). Circadian disruption in experimental cancer processes. Integr. Cancer Ther..

[127] Dong Z., Zhang G., Qu M., Gimple R.C., Wu Q., Qiu Z., Prager B.C., Wang X., Kim L.J., Morton A.R. (2019). Targeting glioblastoma stem cells through disruption of the circadian clock. Cancer Discov..

[128] Yu M., Li W., Wang Q., Wang Y., Lu F. (2018). Circadian regulator NR1D2 regulates glioblastoma cell proliferation and motility. Oncogene.

[129] Srour B., Plancoulaine S., A Andreeva V., Fassier P., Julia C., Galan P., Hercberg S., Deschasaux M., Latino-Martel P., Touvier M. (2018). Circadian nutritional behaviours and cancer risk: New insights from the NutriNet-santé prospective cohort study: Disclaimers. Int. J. Cancer.

[130] Kogevinas M., Espinosa A., Castello A., Gomez-Acebo I., Guevara M., Martin V., Amiano P., Alguacil J., Peiro R., Moreno V. (2018). Effect of mistimed eating patterns on breast and prostate cancer risk (MCC-Spain Study). Int. J. Cancer.

[131] Ruoslahti E. (1996). How cancer spreads. Sci. Am..

[132] Cooper G., Adams K. (2022). The Cell: A Molecular Approach.

[133] Tsuchiya Y., Umemura Y., Yagita K. (2020). Circadian clock and cancer: From a viewpoint of cellular differentiation. Int. J. Urol..

[134] Yang G., Chen L., Grant G.R., Paschos G., Song W.L., Musiek E.S., Lee V., Mcloughlin S.C., Grosser T., Cotsarelis G. (2016). Timing of expression of the core clock gene Bmal1 influences its effects on aging and survival. Sci. Transl. Med..

[135] Langmesser S., Tallone T., Bordon A., Rusconi S., Albrecht U. (2008). Interaction of circadian clock proteins PER2 and CRY with BMAL1 and CLOCK. BMC Mol. Biol..

[136] Kinouchi K., Sassone-Corsi P. (2020). Metabolic rivalry: Circadian homeostasis and tumorigenesis. Nat. Rev. Cancer.

[137] Yu H., Meng X., Wu J., Pan C., Ying X., Zhou Y., Liu Z., Huang W. (2013). Cryptochrome 1 overexpression correlates with tumor progression and poor prognosis in patients with colorectal cancer. PLoS ONE.

[138] Ye Y., Xiang Y., Ozguc F.M., Kim Y., Liu C.J., Park P.K., Hu Q., Diao L., Lou Y., Lin C. (2018). The genomic landscape and pharmacogenomic interactions of clock genes in cancer chronotherapy. Cell Syst..

[139] Cadenas C., Cadenas C., Van De Sandt L., Edlund K., Lohr M., Hellwig B., Marchan R., Schmidt M., Rahnenführer J., Oster H. (2014). Loss of circadian clock gene expression is associated with tumor progression in breast cancer. Cell Cycle.

[140] Mannic T., Meyer P., Triponez F., Pusztaszeri M., Le Martelot G., Mariani O., Schmitter D., Sage D., Jacques Philippe J., Dibner C. (2013). Circadian clock characteristics are altered in human thyroid malignant nodules. J. Clin. Endocrinol. Metab..

[141] Yang X., Wood P.A., Ansell C.M., Quiton D.F.T., Oh E.Y., Du-Quiton J., Hrushesky W.J. (2009). The circadian clock gene Per1 suppresses cancer cell proliferation and tumor growth at specific times of day. Chronobiol. Int..

[142] Kiessling S., Beaulieu-Laroche L., Blum I.D., Landgraf D., Welsh D.K., Storch K.F., Labrecque N., Cermakian N. (2017). Enhancing circadian clock function in cancer cells inhibits tumor growth. BMC Biol..

[143] Johnson D.G., Schneider-Broussard R. (1998). Role of E2F in cell cycle control and cancer. Front. Biosci..

[144] Kumar P.V.A., Dakup P.P., Sarkar S., Modasia J.B., Motzner M.S., Gaddameedhi S. (2019). Focus: Clocks and Cycles: It’s About Time: Advances in Understanding the Circadian Regulation of DNA Damage and Repair in Carcinogenesis and Cancer Treatment Outcomes. Yale J. Biol. Med..

[145] Broustas C.G., Lieberman H.B. (2014). Lieberman, DNA damage response genes and the development of cancer metastasis. Radiat. Res..

[146] Bevinakoppamath S., Ramachandra S.C., Yadav A.K., Basavaraj V., Vishwanath P., Prashant A. (2022). Understanding the emerging link between circadian rhythm, Nrf2 pathway, and breast cancer to overcome drug resistance. Front. Pharmacol..

[147] Sulli G., Lam M.T.Y., Panda S. (2019). Interplay between circadian clock and cancer: New frontiers for cancer treatment. Trends Cancer.

[148] Domoto T., Pyko I.V., Furuta T., Miyashita K., Uehara M., Shimasaki T., Nakada M., Minamoto T. (2016). Glycogen synthase kinase-3β is a pivotal mediator of cancer invasion and resistance to therapy. Cancer Sci..

[149] Ougolkov A.V., Fernandez-Zapico M.E., Savoy D.N., Urrutia R.A., Billadeau D.D. (2005). Glycogen synthase kinase-3beta participates in nuclear factor kappaB-mediated gene transcription and cell survival in pancreatic cancer cells. Cancer Res..

[150] Vincent E.E., Elder D.J.E., O′Flaherty L., Pardo O.E., Dzien P., Phillips L., Morgan C., Pawade J., May M.T., Sohail M. (2014). Glycogen synthase kinase 3 protein kinase activity is frequently elevated in human non-small cell lung carcinoma and supports tumour cell proliferation. PLoS ONE.

[151] Bilim V., Ougolkov A., Yuuki K., Naito S., Kawazoe H., Muto A., Oya M., Billadeau D., Motoyama T., Tomita Y. (2009). Glycogen synthase kinase-3: A new therapeutic target in renal cell carcinoma. Br. J. Cancer.

[152] Ougolkov A.V., Bone N.D., Fernandez-Zapico M.E., Kay N.E., Billadeau D.D. (2007). Inhibition of glycogen synthase kinase-3 activity leads to epigenetic silencing of nuclear factor kappaB target genes and induction of apoptosis in chronic lymphocytic leukemia B cells. Blood.

[153] Fu L., Kettner N.M. (2013). The circadian clock in cancer development and therapy. Prog. Mol. Biol. Transl. Sci..

[154] Haus E.L., Smolensky M.H. (2013). Shift work and cancer risk: Potential mechanistic roles of circadian disruption, light at night, and sleep deprivation. Sleep Med. Rev..

[155] Meek D.W., Anderson C.W. (2009). Posttranslational modification of p53: Cooperative integrators of function. Cold Spring Harb. Perspect. Biol..

[156] Donzelli M., Draetta G.F. (2003). Regulating mammalian checkpoints through Cdc25 inactivation. EMBO Rep..

[157] Sancar A., Lindsey-Boltz L.A., Kang T.H., Reardon J.T., Lee J.H., Ozturk N. (2010). Circadian clock control of the cellular response to DNA damage. FEBS Lett..

[158] Masri S., Sassone-Corsi P. (2018). The emerging link between cancer, metabolism, and circadian rhythms. Nat. Med..

[159] Cheng H.Y., Papp J.W., Varlamova O., Dziema H., Russell B., Curfman J.P., Nakazawa T., Shimizu K., Okamura H., Impey S. (2007). microRNA modulation of circadian-clock period and entrainment. Neuron.

[160] Reszka E., Zienolddiny S. (2018). Epigenetic Basis of Circadian Rhythm Disruption in Cancer. Methods Mol. Biol..

[161] Dunlap J.C. (1999). Molecular bases for circadian clocks. Cell.

[162] Dickmeis T. (2009). Glucocorticoids and the circadian clock. J. Endocrinol..

[163] Cohen S.M., Ellwein L.B. (1990). Cell proliferation in carcinogenesis. Science.

[164] Fu L., Lee C.C. (2003). The circadian clock: Pacemaker and tumour suppressor. Nat. Rev. Cancer.

[165] Lévi F. (2001). Circadian chronotherapy for human cancers. Lancet. Oncol..

[166] Innominato P.F., Roche V.P., Palesh O.G., Ulusakarya A., Spiegel D., Lévi F.A. (2014). The circadian timing system in clinical oncology. Ann. Med..

[167] Imran S.M., Shao G.N., Kim H. (2016). https://www.sciencedirect.com/science/article/abs/pii/S092633731500346X.

[168] Dimitrov S., Lange T., Tieken S., Fehm H.L., Born J. (2014). Sleep associated regulation of T helper 1/T helper 2 cytokine balance in humans. Brain Behav. Immun..

[169] Imeri L., Opp M.R., Krueger J.M. (1993). An IL-1 receptor and an IL-1 receptor antagonist attenuate muramyl dipeptide- and IL-1-induced sleep and fever. Am. J. Physiol..

[170] Neuzillet C., Tijeras-Raballand A., Cohen R., Cros J., Faivre S., Raymond E., de Gramont A. (2015). Targeting the TGFβ pathway for cancer therapy. Pharmacol. Ther..

[171] Walker W.H., Borniger J.C. (2019). Molecular Mechanisms of Cancer-Induced Sleep Disruption. Int. J. Mol. Sci..

[172] Lozano-Lorca M., Olmedo-Requena R., Vega-Galindo M.V., Vázquez-Alonso F., Jiménez-Pacheco A., Salcedo-Bellido I., Sánchez M.J., Jiménez-Moleón J.J. (2020). Night Shift Work, Chronotype, Sleep Duration, and Prostate Cancer Risk: CAPLIFE Study. Int. J. Environ. Res. Public Health.

[173] Markt S.C., Grotta A., Nyren O., Adami H.O., Mucci L.A., Valdimarsdottir U.A., Stattin P., Bellocco R., Lagerros Y.T. (2015). Insufficient Sleep and Risk of Prostate Cancer in a Large Swedish Cohort. Sleep.

[174] Salamanca-Fernández E., Rodríguez-Barranco M., Guevara M., Ardanaz E., Olry de Labry Lima A., Sánchez M.J. (2018). Night-shift work and breast and prostate cancer risk: Updating the evidence from epidemiological studies. An. Sist. Sanit. Navar..

[175] Benna C., Helfrich-Förster C., Rajendran S., Monticelli H., Pilati P., Nitti D., Mocellin S. (2017). Genetic variation of clock genes and cancer risk: A field synopsis and meta-analysis. Oncotarget.

[176] Sancar A., Van Gelder R.N. (2021). Clocks, cancer, and chronochemotherapy. Science.

[177] Peplonska B., Bukowska A., Lie J.A., Gromadzinska J., Zienolddiny S. (2016). Night shift work and other determinants of estradiol, testosterone, and dehydroepiandrosterone sulfate among middle-aged nurses and midwives. Scand. J. Work. Environ. Health.

[178] Szkiela M., Kusideł E., Makowiec-Dąbrowska T., Kaleta D. (2021). How the Intensity of Night Shift Work Affects Breast Cancer Risk. Int. J. Environ. Res. Public Health.

[179] Hurley S., Goldberg D., Von Behren J., Clague DeHart J., Wang S., Reynolds P. (2019). Chronotype and postmenopausal breast cancer risk among women in the California Teachers Study. Chronobiol. Int..

[180] Longcope C. (1990). Relationships of estrogen to breast cancer, of diet to breast cancer, and of diet to estradiol metabolism. J. Natl. Cancer Inst..

[181] Fishman J., Bradlow H.L., Schneider J., Anderson K.E., Kappas A. (1980). Radiometric analysis of biological oxidations in man: Sex differences in estradiol metabolism. Proc. Natl. Acad. Sci. USA.

[182] Fishman J., Martucci C. (1980). Biological properties of 16 alpha-hydroxyestrone: Implications in estrogen physiology and pathophysiology. J. Clin. Endocrinol. Metab..

[183] Bradlow H.L., Hershcopf R.E., Fishman J.F. (1986). Oestradiol 16 alpha-hydroxylase: A risk marker for breast cancer. Cancer Surv..

[184] Swaneck G.E., Fishman J. (1988). Covalent binding of the endogenous estrogen 16 alpha-hydroxyestrone to estradiol receptor in human breast cancer cells: Characterization and intranuclear localization. Proc. Natl. Acad. Sci. USA.

[185] Michnovicz J.J., Hershcopf R.J., Naganuma H., Bradlow H.L., Fishman J. (1986). Increased 2-hydroxylation of estradiol as a possible mechanism for the anti-estrogenic effect of cigarette smoking. N. Engl. J. Med..

[186] Samanta S. (2021). The Potential Oncostatic Effects of Melatonin against Prostate Cancer. Crit. Rev. Oncog..

[187] Baan R., Grosse Y., Straif K., Secretan B., El Ghissassi F., Bouvard V., Benbrahim-Tallaa L., Guha N., Freeman C., Galichet L. (2009). A review of human carcinogens—Part F: Chemical agents and related occupations. Lancet. Oncol..

[188] Samanta S. (2021). A Profound Relationship between Circadian Rhythm Dysfunction and Cancer Progression: An Approach to Exploration. Crit. Rev. Oncog..

[189] Morales-Santana S., Morell S., Leon J., Carazo-Gallego A., Jimenez-Lopez J.C., Morell M. (2019). An Overview of the Polymorphisms of Circadian Genes Associated with Endocrine Cancer. Front. Endocrinol..

[190] Valenzuela F.J., Vera J., Venegas C., Muñoz S., Oyarce S., Muñoz K., Lagunas C. (2016). Evidences of Polymorphism Associated with Circadian System and Risk of Pathologies: A Review of the Literature. Int. J. Endocrinol..

[191] Chu L.W., Zhu Y., Yu K., Zheng T., Yu H., Zhang Y., Sesterhenn I., Chokkalingam A.P., Danforth K.N., Shen M.C. (2008). Variants in circadian genes and prostate cancer risk: A population-based study in China. Prostate Cancer Prostatic Dis..

[192] Chuffa L., Seiva F., Cucielo M., Silveira H., Reiter R.J., Lupi L.A. (2019). Clock genes and the role of melatonin in cancer cells: An overview. Melatonin Res..

[193] Steketee K., Timmerman L., Ziel-van der Made A.C., Doesburg P., Brinkmann A.O., Trapman J. (2002). Broadened ligand responsiveness of androgen receptor mutants obtained by random amino acid substitution of H874 and mutation hot spot T877 in prostate cancer. Int. J. Cancer.

[194] van de Wijngaart D.J., Molier M., Lusher S.J., Hersmus R., Jenster G., Trapman J., Dubbink H.J. (2010). Systematic structure-function analysis of androgen receptor Leu701 mutants explains the properties of the prostate cancer mutant L701H. J. Biol. Chem..

[195] Rimler A., Culig Z., Levy-Rimler G., Lupowitz Z., Klocker H., Matzkin H., Bartsch G., Zisapel N. (2001). Melatonin elicits nuclear exclusion of the human androgen receptor and attenuates its activity. Prostate.

[196] Russo J., Russo I.H. (2006). The role of estrogen in the initiation of breast cancer. J. Steroid Biochem. Mol. Biol..

[197] Toma J.G., Amerongen H.M., Hennes S.C., O‘Brien M.G., McBlain W.A., Buzzell G.R. (1987). Effects of olfactory bulbectomy, melatonin, and/or pinealectomy on three sublines of the Dunning R3327 rat prostatic adenocarcinoma. J. Pineal Res..

[198] Dakubo G.D., Parr R.L., Costello L.C., Franklin R.B., Thayer R. (2006). Altered metabolism and mitochondrial genome in prostate cancer. J. Clin. Pathol..

[199] Costello L.C., Franklin R.B. (2000). The intermediary metabolism of the prostate: A key to understanding the pathogenesis and progression of prostate malignancy. Oncology.

[200] Hevia D., Sainz R.M., Blanco D., Quirós I., Tan D.X., Rodríguez C., Mayo J.C. (2008). Melatonin uptake in prostate cancer cells: Intracellular transport versus simple passive diffusion. J. Pineal Res..

[201] Green M.M., Hiley C.T., Shanks J.H., Bottomley I.C., West C.M., Cowan R.A., Stratford I.J. (2007). Expression of vascular endothelial growth factor (VEGF) in locally invasive prostate cancer is prognostic for radiotherapy outcome. Int. J. Radiat. Oncol. Biol. Phys..

[202] Hevia D., González-Menéndez P., Quiros-González I., Miar A., Rodríguez-García A., Tan D.X., Reiter R.J., Mayo J.C., Sainz R.M. (2015). Melatonin uptake through glucose transporters: A new target for melatonin inhibition of cancer. J. Pineal Res..

[203] Shen D., Ju L., Zhou F., Yu M., Ma H., Zhang Y., Liu T., Xiao Y., Wang X., Qian K. (2021). The inhibitory effect of melatonin on human prostate cancer. Cell Commun. Signal..

[204] Duque J.L.F., Loughlin K.R., Adam R.M., Kantoff P.W., Zurakowski D., Freeman M.R. (1999). Plasma levels of vascular endothelial growth factor are increased in patients with metastatic prostate cancer. Urology.

[205] Samanta S. (2018). Hypoxia inducible factor-1 (HIF-1) and cancer progression: A comprehensive review. Indian J. Cancer Edu. Res..

[206] Iesanu M.I., Zahiu C.D.M., Dogaru I.A., Chitimus D.M., Pircalabioru G.G., Voiculescu S.E., Isac S., Galos F., Pavel B., O’Mahony S.M. (2022). Melatonin–Microbiome two-sided interaction in Dysbiosis-associated conditions. Antioxidants.

[207] Ozturk N., Ozturk D., Halil Kavakli I., Okyar A. (2017). Molecular aspects of circadian pharmacology and relevance for cancer chronotherapy. Int. J. Mol. Sci..

[208] Dallmann R., Okyar A., Lévi F. (2016). Dosing-time makes the poison: Circadian regulation and pharmacotherapy. Trends Mol. Med..

[209] Antoch M.P., Kondratov R.V., Takahashi J.S. (2005). Circadian clock genes as modulators of sensitivity to genotoxic stress. Cell Cycle.

[210] Hu J., Lieb J.D., Sancar A., Adar S. (2016). Cisplatin DNA damage and repair maps of the human genome at single-nucleotide resolution. Proc. Natl. Acad. Sci. USA.

[211] Yang Y., Liu Z., Selby C.P., Sancar A. (2019). Long-term, genome-wide kinetic analysis of the effect of the circadian clock and transcription on the repair of cisplatin-DNA adducts in the mouse liver. J. Biol. Chem..

[212] Kang T.H., Lindsey-Boltz L.A., Reardon J.T., Sancar A. (2010). Circadian control of XPA and excision repair of cisplatin-DNA damage by cryptochrome and HERC2 ubiquitin ligase. Proc. Natl. Acad. Sci. USA.

[213] Dakup P.P., Porter K.I., Little A.A., Gajula R.P., Zhang H., Skornyakov E., Kemp M.G., Van Dongen H.P.A., Gaddameedhi S. (2018). The circadian clock regulates cisplatin-induced toxicity and tumor regression in melanoma mouse and human models. Oncotarget.

[214] Hashikawa K.I., Katamune C., Kusunose N., Matsunaga N., Koyanagi S., Ohdo S. (2017). Dysfunction of the circadian transcriptional factor CLOCK in mice resists chemical carcinogen-induced tumorigenesis. Sci. Rep..

[215] Huisman S.A., Oklejewicz M., Ahmadi A.R., Tamanini F., Ijzermans J.N., van der Horst G.T., de Bruin R.W. (2015). Colorectal liver metastases with a disrupted circadian rhythm phase shift the peripheral clock in liver and kidney. Int. J. Cancer.

[216] Van Dycke K.C., Rodenburg W., van Oostrom C.T., Van Kerkhof L.W., Pennings J.L., Roenneberg T., van Steeg H., van der Horst G. (2015). TChronically alternating light cycles increase breast cancer risk in mice. Curr. Biol..

[217] Comas M., Kuropatwinski K.K., Wrobel M., Toshkov I., Antoch M.P. (2014). Daily rhythms are retained both in spontaneously developed sarcomas and in xenografts grown in immunocompromised SCID mice. Chronobiol. Int..

[218] Huang C., Zhang C., Cao Y., Li J., Bi F. (2023). Major roles of the circadian clock in cancer. Cancer Biol. Med..

[219] He B., Nohara K., Park N., Park Y.S., Guillory B., Zhao Z., Chen Z. (2016). The small molecule nobiletin targets the molecular oscillator to enhance circadian rhythms and protect against metabolic syndrome. Cell Metab..

[220] Lee Y., Field J.M., Sehgal A. (2021). Circadian rhythms, disease and chronotherapy. J. Biol. Rhythm..

[221] Dong D., Yang D., Lin L., Wang S., Wu B. (2020). Circadian rhythm in pharmacokinetics and its relevance to chronotherapy. Biochem. Pharmacol..

[222] Lu D., Wang Z., Wu B. (2022). Pharmacokinetics-based chronotherapy. Curr. Drug Metab..

[223] Miro C., Docimo A., Barrea L., Verde L., Cernea S., Sojat A.S., Marina L.V., Docimo G., Colao A., Dentice M. (2023). “Time” for obesity-related cancer: The role of the circadian rhythm in cancer pathogenesis and treatment. Semin. Cancer Biol..

[224] García-Costela M., Escudero-Feliú J., Puentes-Pardo J.D., San Juán S.M., Morales-Santana S., Ríos-Arrabal S., Carazo A., León J. (2020). Circadian genes as therapeutic targets in pancreatic cancer. Front. Endocrinol..

[225] Dose B., Yalçin M., Dries S.P., Relógio A. (2023). TimeTeller for timing health: The potential of circadian medicine to improve performance, prevent disease and optimize treatment. Front. Digit. Health.

[226] Del Prete A., Salvi V., Soriani A., Laffranchi M., Sozio F., Bosisio D., Sozzani S. (2023). Dendritic cell subsets in cancer immunity and tumor antigen sensing. Cell. Mol. Immunol..

[227] Talaat I.M., Elemam N.M., Zaher S., Saber-Ayad M. (2022). Checkpoint molecules on infiltrating immune cells in colorectal tumor microenvironment. Front. Med..

[228] Kamali A.N., Bautista J.M., Eisenhut M., Hamedifar H. (2023). Immune checkpoints and cancer immunotherapies: Insights into newly potential receptors and ligands. Ther. Adv. Vaccines Immunother..

[229] Mirian M., Hariri A., Yadollahi M., Kohandel M. (2022). Circadian and immunity cycle talk in cancer destination: From biological aspects to in silico analysis. Cancers.

[230] Zhang Z., Zeng P., Gao W., Zhou Q., Feng T., Tian X. (2021). Circadian clock: A regulator of the immunity in cancer. Cell Commun. Signal..

[231] Zhou J., Wang J., Zhang X., Tang Q. (2021). New insights into cancer chronotherapies. Front. Pharmacol..

[232] Qu M. (2023). Molecular crosstalk between circadian clock and cancer and therapeutic implications. Front. Nutr..

[233] Altman B.J., Hsieh A.L., Sengupta A., Krishnanaiah S.Y., Stine Z.E., Walton Z.E., Gouw A.M., Venkataraman A., Li B., Goraksha-Hicks P. (2015). MYC disrupts the circadian clock and metabolism in cancer cells. Cell Metab..

[234] Relogio A., Thomas P., Medina-Perez P., Reischl S., Bervoets S., Gloc E., Riemer P., Mang-Fatehi S., Maier B., Schäfer R. (2014). Ras-mediated deregulation of the circadian clock in cancer. PLoS Genet..

[235] Gutiérrez-Monreal M.A., Treviño V., Moreno-Cuevas J.E., Scott S.P. (2016). Identification of circadian-related gene expression profiles in entrained breast cancer cell lines. Chronobiol. Int..

[236] Selfridge J.M., Gotoh T., Schiffhauer S., Liu J., Stauffer P.E., Li A., Capelluto D.G.S., Finkielstein C.V. (2016). Chronotherapy: Intuitive, sound, founded… but not broadly applied. Drugs.

[237] Ballesta A., Innominato P.F., Dallmann R., Rand D.A., Lévi F.A. (2017). Systems chronotherapeutics. Pharmacol. Rev..

[238] Zhu X., Maier G., Panda S. (2023). Learning from circadian rhythm to transform cancer prevention, prognosis, and survivorship care. Trends Cancer.

[239] Yang Y., Xu T., Zhang Y., Qin X. (2017). Molecular basis for the regulation of the circadian clock kinases CK1δ and CK1ε. Cell. Signal..

[240] Kennaway D.J., Varcoe T.J., Voultsios A., Salkeld M.D., Rattanatray L., Boden M.J. (2015). Acute inhibition of casein kinase 1δ/ε rapidly delays peripheral clock gene rhythms. Mol. Cell. Biochem..

[241] Rosenberg L.H., Lafitte M., Quereda V., Grant W., Chen W., Bibian M., Noguchi Y., Fallahi M., Yang C., Chang J.C. (2015). Therapeutic targeting of casein kinase 1δ in breast cancer. Sci. Transl. Med..

[242] Oshima T., Niwa Y., Kuwata K., Srivastava A., Hyoda T., Tsuchiya Y., Kumagai M., Tsuyuguchi M., Tamaru T., Sugiyama A. (2019). Cell-based screen identifies a new potent and highly selective CK2 inhibitor for modulation of circadian rhythms and cancer cell growth. Sci. Adv..

[243] Zhao X., Hirota T., Han X., Cho H., Chong L.W., Lamia K., Liu S., Atkins A.R., Banayo E., Liddle C. (2016). Circadian amplitude regulation via FBXW7-targeted REV-ERBα degradation. Cell.

[244] Gao J., Azmi A.S., Aboukameel A., Kauffman M., Shacham S., Abou-Samra A.B., Mohammad R.M. (2014). Nuclear retention of Fbw7 by specific inhibitors of nuclear export leads to Notch1 degradation in pancreatic cancer. Oncotarget.

[245] Huang H.L., Weng H.Y., Wang L.Q., Yu C.H., Huang Q.J., Zhao P.P., Wen J.Z., Zhou H., Qu L.H. (2012). Triggering Fbw7-mediated proteasomal degradation of c-Myc by oridonin induces cell growth inhibition and apoptosis. Mol. Cancer Ther..

[246] Lauvrak S.U., Munthe E., Kresse S.H., Stratford E.W., Namløs H.M., Meza-Zepeda L.A., Myklebost O. (2013). Functional characterisation of osteosarcoma cell lines and identification of mRNAs and miRNAs associated with aggressive cancer phenotypes. Br. J. Cancer.

[247] Schopf F.H., Biebl M.M., Buchner J. (2017). The HSP90 chaperone machinery. Nat. Rev. Mol. Cell Biol..

[248] Hoter A., El-Sabban M.E., Naim H.Y. (2018). The HSP90 family: Structure, regulation, function, and implications in health and disease. Int. J. Mol. Sci..

[249] Wu B.X., Hong F., Zhang Y., Ansa-Addo E., Li Z. (2016). GRP94/gp96 in cancer: Biology, structure, immunology, and drug development. Adv. Cancer Res..

[250] Hu X., Liu X., Moisan J., Wang Y., Lesch C.A., Spooner C., Morgan R.W., Zawidzka E.M., Mertz D., Bousley D. (2016). Synthetic RORγ agonists regulate multiple pathways to enhance antitumor immunity. Oncoimmunology.

[251] Hu X., Majchrzak K., Liu X., Wyatt M.M., Spooner C.J., Moisan J., Zou W., Carter L.L., Paulos C.M. (2018). In vitro priming of adoptively transferred T cells with a RORγ agonist confers durable memory and stemness in vivo. Cancer Res..

[252] Battaglin F., Chan P., Pan Y., Soni S., Qu M., Spiller E.R., Castanon S., Torres E.T.R., Mumenthaler S.M., Kay S.A. (2021). Clocking cancer: The circadian clock as a target in cancer therapy. Oncogene.

[253] Mormont M.C., Levi F. (2003). Cancer chronotherapy: Principles, applications, and perspectives. Cancer Interdiscip. Int. J. Am. Cancer Soc..

[254] Lévi F. (2002). From circadian rhythms to cancer chronotherapeutics. Chronobiol. Int..

